# Maps of Open Chromatin Guide the Functional Follow-Up of Genome-Wide Association Signals: Application to Hematological Traits

**DOI:** 10.1371/journal.pgen.1002139

**Published:** 2011-06-30

**Authors:** Dirk S. Paul, James P. Nisbet, Tsun-Po Yang, Stuart Meacham, Augusto Rendon, Katta Hautaviita, Jonna Tallila, Jacqui White, Marloes R. Tijssen, Suthesh Sivapalaratnam, Hanneke Basart, Mieke D. Trip, Consortium Cardiogenics, Consortium MuTHER, Berthold Göttgens, Nicole Soranzo, Willem H. Ouwehand, Panos Deloukas

**Affiliations:** 1 Wellcome Trust Sanger Institute, Hinxton, United Kingdom; 2 Department of Haematology, University of Cambridge, Cambridge, United Kingdom; 3 National Health Service Blood and Transplant (NHSBT), Cambridge, United Kingdom; 4 Biostatistics Unit, Medical Research Council, Cambridge, United Kingdom; 5 Cambridge Institute for Medical Research, University of Cambridge, Cambridge, United Kingdom; 6 Department of Vascular Medicine, Academic Medical Centre Amsterdam, Amsterdam, The Netherlands; 7 Department of Twin Research and Genetic Epidemiology, King's College London, London, United Kingdom; The University of North Carolina at Chapel Hill, United States of America

## Abstract

Turning genetic discoveries identified in genome-wide association (GWA) studies into biological mechanisms is an important challenge in human genetics. Many GWA signals map outside exons, suggesting that the associated variants may lie within regulatory regions. We applied the formaldehyde-assisted isolation of regulatory elements (FAIRE) method in a megakaryocytic and an erythroblastoid cell line to map active regulatory elements at known loci associated with hematological quantitative traits, coronary artery disease, and myocardial infarction. We showed that the two cell types exhibit distinct patterns of open chromatin and that cell-specific open chromatin can guide the finding of functional variants. We identified an open chromatin region at chromosome 7q22.3 in megakaryocytes but not erythroblasts, which harbors the common non-coding sequence variant rs342293 known to be associated with platelet volume and function. Resequencing of this open chromatin region in 643 individuals provided strong evidence that rs342293 is the only putative causative variant in this region. We demonstrated that the C- and G-alleles differentially bind the transcription factor EVI1 affecting *PIK3CG* gene expression in platelets and macrophages. A protein–protein interaction network including up- and down-regulated genes in *Pik3cg* knockout mice indicated that *PIK3CG* is associated with gene pathways with an established role in platelet membrane biogenesis and thrombus formation. Thus, rs342293 is the functional common variant at this locus; to the best of our knowledge this is the first such variant to be elucidated among the known platelet quantitative trait loci (QTLs). Our data suggested a molecular mechanism by which a non-coding GWA index SNP modulates platelet phenotype.

## Author Summary

Genome-wide scans have revealed multiple genetic regions underlying complex traits. However, the transition from an initial association signal to identifying the functional DNA change(s) has proved challenging. Many of the DNA changes discovered are located outside protein-coding regions and may exert their effects through gene regulation. We screened genetic regions associated with hematological traits in erythroblasts (red blood cells) and megakaryocytes (platelet-producing cells) and mapped sites of open chromatin, which harbor active gene regulatory elements. We investigated a DNA sequence change located within a site of open chromatin at chromosome 7 in megakaryocytes, but not erythroblasts, known to be associated with platelet volume. We showed that this DNA change is functional due to alteration of the binding site of a transcription factor, which regulates the expression of a gene that affects platelet characteristics. Mice lacking this gene revealed significant differences in expression of several important platelet genes compared to wild-type mice. The approach described here can be applied in different cell types to functionally follow-up association signals with many other biological traits by identification of the causative base change and how it affects gene function, thus paving the way to clinical benefit.

## Introduction

In recent years, genome-wide association (GWA) studies have driven the discovery of genetic loci associated with a multitude of complex traits and diseases. However, due to linkage disequilibrium (LD), the single-nucleotide polymorphisms (SNPs) assayed in large-scale GWA studies typically yield a proxy for the actual causative variant(s) and often fail to even pinpoint the underlying gene [1]–[3]. Therefore, despite great success in biological discovery, GWA studies have shed little light on which are the functional variants, common and/or low-frequency, and how they relate to biological mechanisms.

Several strategies have been proposed to identify causative sequence variants underlying GWA signals, including fine-mapping (i.e. targeted resequencing followed by additional genotyping of large cohorts) and functional annotation using biochemical assays (e.g. gene expression studies) [1], [3]. Since many initial association signals are localized at intronic and intergenic regions, it has been postulated that they may correspond to gene regulatory variants [4]. Therefore, functional variants may exert their effects through regulation of gene expression, which can vary substantially among tissues and cell types [5], [6].

Integrated studies have illustrated how particular trait-associated alleles are mechanistically relevant to cellular functions and pathways [7]–[12]. In a recent study, Gaulton et al. used the formaldehyde-assisted isolation of regulatory elements (FAIRE) technique to identify pancreatic islet-specific open chromatin regions, some of which embed known diabetes risk variants [13]. FAIRE is an elegant approach to isolating nucleosome-depleted regions (NDRs) that encompass active regulatory elements, and thus, to accessing regulatory variants [14], [15].

Heritable hematological traits are of particular interest in studying the architecture of complex traits, since relevant cell types for functional assays are easily accessible. In addition, genetic loci associated with platelet counts, *SH2B3–ATXN2* and *PTPN11*, have been reported to be associated with coronary artery disease (CAD) and myocardial infarction (MI) suggesting a possible role for platelets as an intermediate phenotype [16].

Here we describe maps of open chromatin generated by FAIRE in a megakaryocytic and an erythroblastoid cell line at known GWA loci associated with hematological quantitative traits, CAD and MI. We report substantial differences in chromatin architecture among the two cell types and assess the phenotypic causality of variants that fall into NDRs specific to either cell type. We demonstrate the procedure with the locus at chromosome 7q22.3, which is associated with mean platelet volume and function, where we identify a site of open chromatin in megakaryocytes but not erythroblasts and elucidate the molecular mechanism that contributes to the platelet phenotype.

## Results

### Open chromatin profiles in a megakaryocytic and an erythroblastoid cell line

We profiled chromatin at 62 non-redundant genetic loci representing all known associations, as of November 2009, with 11 cardiovascular traits (Table 1) in a megakaryocytic (MK cells) and an erythroblastoid cell line (EB cells). Maps of open chromatin were created with FAIRE, applying two different formaldehyde cross-linking times and subsequent hybridization to a custom 385,000-oligonucleotide array spanning the selected loci (Materials and Methods). Open chromatin regions showed high concordance across cross-linking conditions, with 95.3% and 93.1% overlapping regions in MK and EB cells, respectively. To achieve higher stringency, we retained only concordant open chromatin regions in each cell type for further analysis (Figure S1). At the interrogated loci, we identified 254 and 251 NDRs in MK and EB cells, respectively (Table S1A), of which 147 (57.9% and 58.6%, respectively) were common to both cell types. FAIRE peak density (per megabase) in our data set is consistent with that in foreskin fibroblast cells reported by the ENCODE Project [17] (Table S2).

**Table 1:** Summary of the genetic loci included on the custom DNA tiling array.

Complex trait	Number of genetic loci	Genomic footprint
Coronary artery disease (CAD) and (early-onset) myocardial infarction (MI)	18	3,605.2 kb
Mean platelet volume (MPV)	12	1,789.7 kb
Platelet counts (PLT)	4	712.2 kb
Platelet signaling (PLS)	15	1,968.4 kb
White blood cell counts (WBC)	1	73.2 kb
Red blood cell counts (RBC)	1	50.0 kb
Mean corpuscular volume (MCV)	4	436.0 kb
Mean corpuscular hemoglobin (MCH)	1	100.0 kb
Systolic blood pressure (SBP)	6	940.0 kb
Diastolic blood pressure (DBP)	8	1,475.0 kb
Hypertension (HYP)	2	240.0 kb
**Total**	**72**	**11,149.7 kb**
**Total unique**	**62**	**9,593.5 kb**

A detailed list, including genomic coordinates of the intervals and references, is presented in Table S9A.

### Characterization of open chromatin regions in relation to gene annotations and cell types

We then analyzed the 254 and 251 NDRs in MK and EB cells, respectively, in context of their genomic location (intergenic, intronic, overlap 5′-untranslated region (UTR), overlap 3′-UTR or exonic; Table S3). We note that our observations are based on a selected set of loci and therefore cannot be extrapolated to the whole genome. NDRs were most frequently located at non-coding segments (98.2% and 92.5% of peaks found only in MK and EB cells, respectively, and 98.1% of peaks common to both cell types). Promoter/5′-UTR regions were enriched in NDRs common to both cell types (28.4%) compared to open chromatin specific to either cell type, namely 4.6% (6.2-fold) and 5.6% (5.1-fold) for MK and EB cells, respectively.

At the interrogated loci, NDRs clustered around transcription start sites (TSS). In MK and EB cells, 70.5% and 76.1% of all FAIRE peaks were located within 20 kb of a TSS, respectively (Figure S2). However, accessible chromatin regions as far as 264 kb upstream of a TSS were also detected (*TBX3* gene locus). Open chromatin observed in MK but not EB cells was located on average 2.80 kb upstream of a TSS. NDRs found in EB but not MK cells were located on average 1.77 kb, whereas NDRs common to both cell types were located on average 0.98 kb upstream of a TSS.

To assess the cell type specificity of NDRs marked by FAIRE in our experiment, we determined the number of peaks in lineage-specific genes for MK and EB cells present on the array (Figure S3; Table S1B). A significant enrichment of FAIRE peaks at MK lineage-specific genes was observed in MK cells, when compared to the number of peaks in EB cells (Wilcoxon rank-sum test, *P*=0.0225). A similar trend of enrichment was observed in EB lineage-specific genes in EB cells (*P*=0.0781). This result highlights the importance of studying chromatin architecture and gene regulatory circuits in a cell type-dependent manner.

### Seven sequence variants associated with cardiovascular-related quantitative traits are located in open chromatin

At seven of the 62 tested loci, we found SNPs in strong LD with the corresponding GWA index SNP (r^2^≥0.8, Phase II HapMap, CEU population) located within a NDR (Table 2). Five out of the seven loci are associated with platelet-related quantitative trait loci (QTLs). We compared the position of sites of open chromatin and sites were either found only in MK but not EB cells (‘MK-specific’, n=2), in EB but not MK cells (‘EB-specific’, n=1) or in both cell types (n=4). The two MK-specific NDRs harboring SNPs associated with mean platelet volume (MPV) are located at an intergenic region of the *FLJ36031–PIK3CG* gene locus (Figure S4A) and an intronic region of *DNM3* (Figure S4B). Both genes, *PIK3CG* and *DNM3*, are upregulated in megakaryocytes as compared to erythroblasts (2.08- and 6.42-fold, respectively, according to the HaemAtlas, a systematic analysis of expression profiles in differentiated human blood cells) [18]. The EB-specific NDR is located at an intergenic region of the *HBS1L–MYB* gene cluster (Figure S4C). Sequence variants at this locus are known to be associated with mean corpuscular volume (MCV) of erythrocytes, mean corpuscular hemoglobin (MCH) and red blood cell counts (RBC). *HBS1L* and *MYB* are upregulated in EB cells (1.30- and 2.40-fold, respectively, according to the HaemAtlas). At the four NDRs common to both cell types, we found variants associated with: platelet signaling (PLS) located at the promoter regions of *PEAR1* (Figure S4D) and *RAF1* (Figure S4E); MPV found at an intronic region of *TMCC2* (Figure S4F); and systolic blood pressure (SBP) at an intronic region of *C10orf32* (*CYP17A1* gene cluster; Figure S4G). Expression profiles of these genes, based on the HaemAtlas data, confirmed transcription in both MK and EB cells (Figure S5A).

**Table 2:** Seven sequence variants associated with cardiovascular-related and hematological intermediate traits are located in nucleosome-depleted regions (NDRs).

Cell type	Trait	Locus	SNP in open chromatin			GWA index SNP		
			ID	MAF	Annotation	ID	r^2^	Distance
MK	MPV	*FLJ36031–PIK3CG*	rs342293	0.45	Intergenic	rs342293	1.00	(index SNP)
MK	MPV	*DNM3*	rs2038479	0.16	Intronic	rs10914144	0.94	10 kb
EB	MCV	*HBS1L–MYB*	rs7775698	0.22	Intergenic	rs9402686	0.85	9 kb
EB/MK	MPV	*TMCC2*	rs1172147	0.35	Intronic	rs1668873	0.89	10 kb
EB/MK	PLS	*PEAR1*	rs4661069	0.11	Promoter	rs3737224	0.83	17 kb
EB/MK	PLS	*RAF1*	rs3806661	0.30	Promoter	rs3729931	0.85	79 kb
EB/MK	SBP	*CYP17A1–C10orf32*	rs3824754	0.07	Intronic	rs1004467	1.00	19 kb

MK: megakaryocytic cell line; EB: erythroblastoid cell line; MPV: mean platelet volume; MCV: mean corpuscular volume of erythrocytes; PLS: platelet signaling; SBP: systolic blood pressure; MAF: minor allele frequency.

### Functional follow-up of the platelet volume and function locus at chromosome 7q22.3

**Figure 1:**
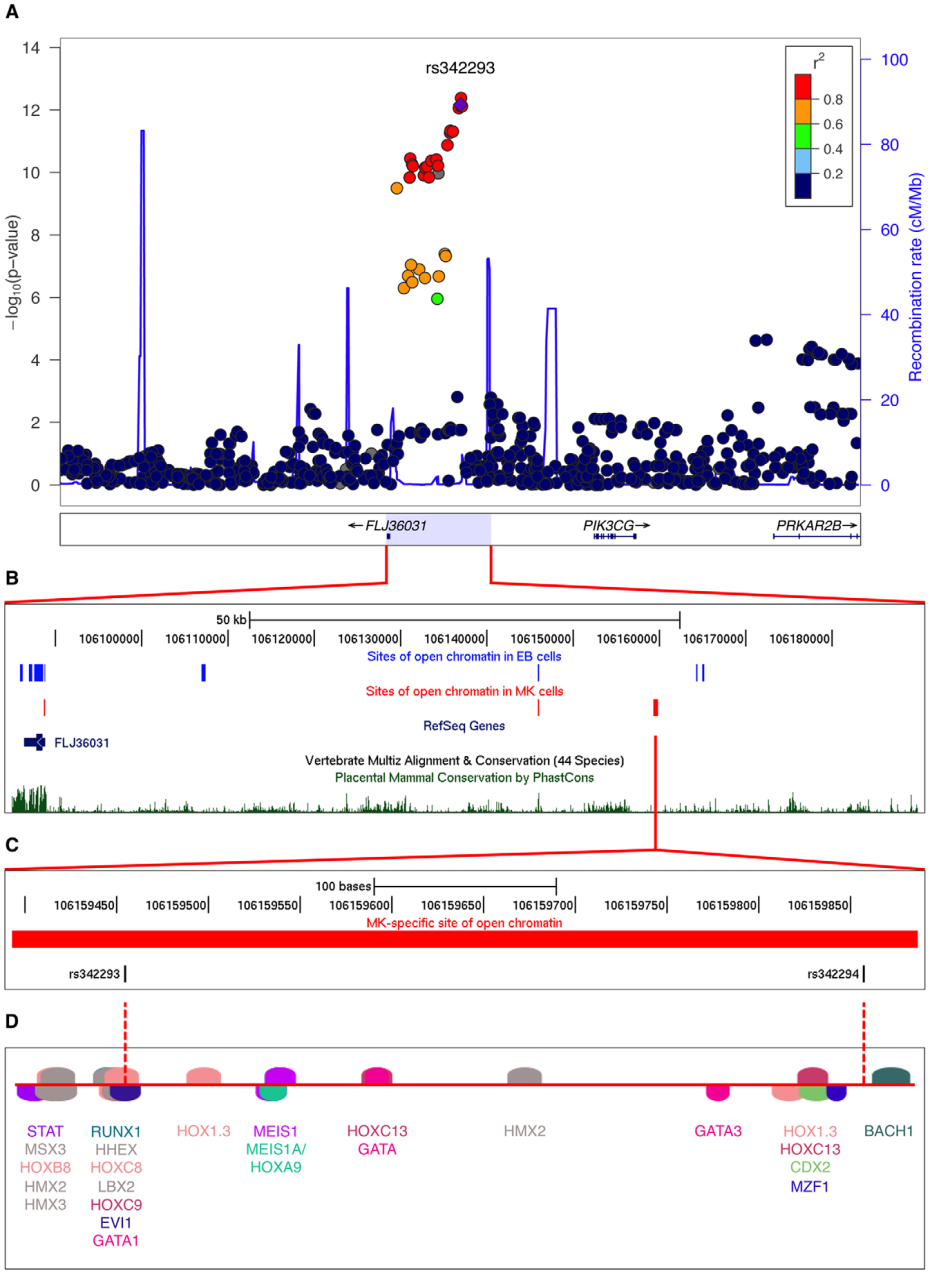
Functional follow-up of the 7q22.3 locus associated with platelet volume and function. (A) Regional plot of the 7q22.3 locus showing data from the discovery GWA meta-analysis as reported by Soranzo et al. The SNP rs342293 is indicated in purple (*P*=6.75×10^−13^).Values for r^2^ are based on the 1000 Genomes Project (Pilot 1, CEU). The data was plotted with LocusZoom v1.1 (http://csg.sph.umich.edu/locuszoom/). (B) Sites of open chromatin marked by FAIRE across the locus in the CHRF-288-11 (MK) and K562 (EB) cell lines (data uploaded as UCSC Genome Browser custom tracks). (C) Site of open chromatin found in MK but not EB cells harboring the common variants rs342293 and rs342294. (D) *In silico* annotation of transcription factor binding sites at the open chromatin region described in (C).The predicted binding events are shown as transcription factor matrices (MatInspector v8.01). Note that some predicted binding sites are overlapping and not visible.

Cell-specific open chromatin regions are likely to play a regulatory role in modulating gene expression in a cell-specific manner. Sequence variants in such NDRs have the potential to impact cell-specific traits, for example MPV. As a proof of principle, we investigated the 65-kb locus associated with mean platelet volume and function at chromosome 7q22.3 (Figure 1A), which harbors a MK-specific NDR as described above. The recombination interval exhibited a total of six distinct NDRs (Figure 1B), two of which are common to both cell types located at an evolutionary conserved element 10 kb upstream of the GWA index SNP rs342293 and at the promoter region of *FLJ36031*, three of which are specific to EB cells and one of which is specific to MK cells. The latter NDR contained SNPs associated with MPV: rs342293 (MAF=0.48, Phase II+III HapMap, CEU) and its best proxy rs342294 (r^2^=1.0, MAF=0.48, Phase II+III HapMap, CEU). Data from the 1000 Genomes Project [19] (Pilot 1, CEU) revealed 34 SNPs in LD (r^2^≥0.8) with rs342293, and confirmed that only rs342293 and rs342294 fall into the MK-specific NDR. This NDR was absent in both FAIRE-chip data sets (8 and 12 min formaldehyde cross-linking conditions) in EB cells (Figure 1C). Irrespective of LD to rs342293, there are no sequence variants reported by the 1000 Genomes Project (Pilot 1, CEU) within sites of open chromatin at the recombination interval in MK cells.

Next, we performed an *in silico* analysis of transcription factor binding sites at the 7q22.3 locus (Table S4). Of the 34 SNPs in LD with rs342293, four (rs342240, rs342247, rs342292 and rs342293) disrupt an *in silico* predicted transcription factor binding site. However, only rs342293 is located within an experimentally verified site of open chromatin in MK cells. Among these four SNPs, rs342293 is the most strongly associated with mean platelet volume [16].

**Figure 2:**
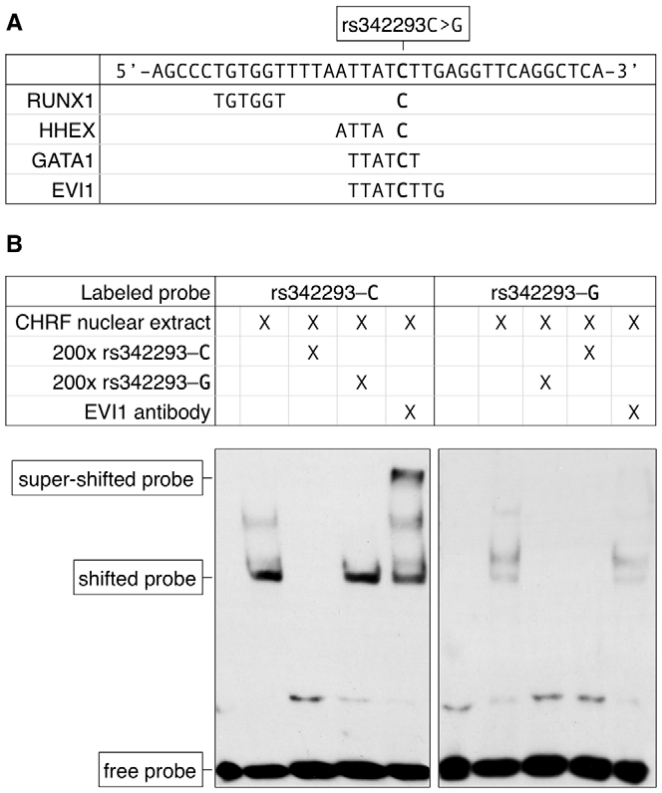
The effect of rs342293C>G on transcription factor binding. (A) Nucleotide sequence of the synthetic probe harboring rs342293 and *in silico* prediction of transcription factor binding sites. The major (C-) allele of rs342293 is shown in bold. The minor (G-) allele of rs342293 is predicted to disrupt the binding motifs of the transcription factors GATA1 (− strand) and EVI1 (−). RUNX1 (+) and HHEX (+) transcription factor binding sites are predicted to be located 9 bp and 1 bp apart from rs342293, respectively. (B) Electrophoretic mobility shift assays (EMSA) in nuclear extracts from the megakaryocytic cell line CHRF-288-11 showed differential binding of the two alleles of rs342293. Competition reactions were performed with a 200-fold molar excess of unlabeled probes. Only the probe harboring the reference allele was able to shift the protein-DNA complex when incubating with EVI1 antibodies. Supershift experiments using GATA1 and RUNX1 antibodies are shown in Figure S6.

The SNP rs342293 is located within overlapping predicted binding sites for the transcription factors BARX2, EVI1, GATA1, HHEX, HOXC8, HOXC9 and LBX2 (Figure 1D). Based on the HaemAtlas data, of these seven transcription factors only EVI1, GATA1 and HHEX are expressed in megakaryocytes (Figure S5B). The C>G substitution leads to disruption of the predicted binding sites of EVI1 and GATA1 (Figure 2A). It is worth noting that *in silico* analysis predicted a RUNX1 transcription factor binding site only 5 bp apart from the EVI1-like binding site.

To obtain the full spectrum of sequence variation at this region, we sequenced the NDR (chr7:106,159,393–106,159,887; 494 bp) in 643 healthy individuals. No other common or low-frequency variants were detected, although a rare, possibly private, SNP was detected in one individual as a heterozygous position (chr7:106,159,601; A>C) located in a polyA-region (Table S5). Based on the above evidence and the absence of any additional common or low-frequency sequence variant at the MK-specific NDR, rs342293 remains as the only likely putative functional candidate underlying the MPV association signal at the 7q22.3 locus. However, it cannot be excluded that additional functional variants may exist outside NDRs and exert their function through, for example disruption of a yet to be identified transcription factor binding site or modulation of DNA methylation.

### The alleles of rs342293 differentially bind the transcription factor EVI1

We then performed electrophoretic mobility shift assays (EMSA) in nuclear extracts from the megakaryocytic cell line CHRF-288-11. We observed a band shift with probes surrounding rs342293 for both the ancestral allele rs342293-C and the alternative allele rs342293-G (Figure 2B). However, the bands were unequally shifted suggesting differential protein binding properties at this position depending on the allele of rs342293. The specific unlabeled competitors supported specificity of the retarded bands. Further, our results suggested superior protein binding to the probe containing the C-allele. With supershift experiments using an EVI1 antibody, we confirmed binding of EVI1 to probes containing the ancestral C-allele, but not to those harboring the alternative G-allele. Supershift experiments with a GATA1 antibody did not support *in vitro* binding of GATA1 transcription factors to this site. We also confirmed RUNX1 transcription factor binding *in vitro* by demonstrating a supershift for both probes with a RUNX1 antibody (Figure S6). We validated these findings by performing chromatin immunoprecipitation combined with next-generation DNA sequencing (ChIP-seq) with GATA1 and RUNX1 antibodies in primary human megakaryocytes. We observed no significant GATA1 but weak RUNX1 binding at this locus further corroborating the EMSA results (Figure S7).

### The SNP rs342293 is associated with *PIK3CG* transcript levels in platelets and macrophages

**Figure 3:**
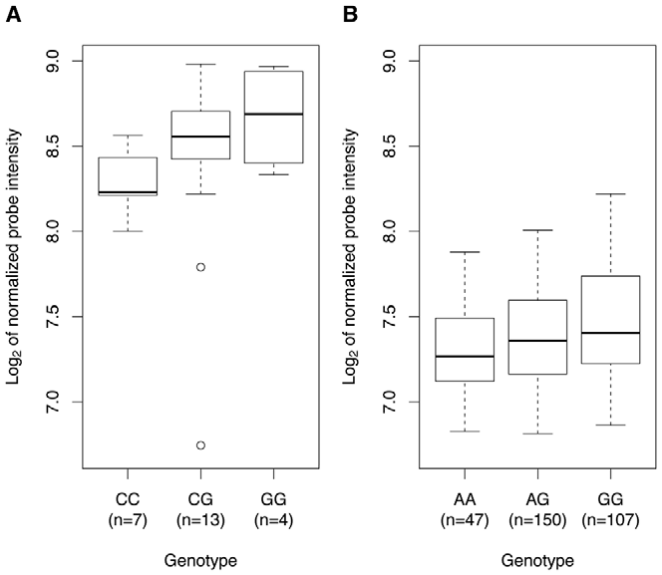
Association of rs342293 genotypes with *PIK3CG* transcript levels in platelets and macrophages. In platelets (A) and macrophages (B), we observed a genotypic effect on *PIK3CG* transcript levels (*P*=0.0542 and *P*=0.0018, respectively). The associations are presented as box-and-whisker plots. In macrophages, the proxy-SNP rs342275A>G (r^2^=0.94, Phase II HapMap, CEU) was used for the eQTL analysis.

Soranzo et al. investigated the association of rs342293 with transcript levels of all known genes within 1 Mb of the GWA index SNP (Figure S5C) in platelets and reported a weak expression QTL (eQTL) association with *PIK3CG* transcript levels (permutation *P*=0.047) [20]. We replicated this finding in an independent sample cohort of 24 healthy individuals showing the same genotypic effect (*P*=0.0542; Figure 3A). In both sample cohorts, none of the other genes within the 1-Mb interval, *PRKAR2B*, *HBP1*, *PBEF1* and *COG5*, had a statistically significant eQTL with rs342293 (*P*>0.1; data not shown). Next, we assessed the *PIK3CG*-eQTL in three different types of white blood cells, macrophages, monocytes and B cells (lymphoblastoid cell line, LCLs), as well as in different tissues, fat and skin (Table S6). We observed a genotypic effect on *PIK3CG* transcript abundance in macrophages (*P*=0.0018; Figure 3B), but not in monocytes and LCLs. Further, we did not observe an association in fat and skin tissues. Our data strengthened the evidence of rs342293 modulating *PIK3CG* transcript levels in platelets and showed that this eQTL is also present in macrophages.

### Gene expression profiles in whole blood of *Pik3cg* ^−/−^ mice

To further our understanding of the role of PIK3CG in platelets, we performed whole-genome gene expression profiling in whole blood of *Pik3cg* knockout mice. We identified 220 differentially expressed genes between knockout (n=3) and wild type mice (n=3) with a fold-change of at least ±1.5 (Table S7). Functional ontology classification of these genes (Table S8) revealed enrichment for ‘regulation of biological characteristic’, e.g. cell size and volume (GO term: 0065008; *P*=2.97×10^−14^), and ‘blood coagulation’ (GO term: 0007596; *P*=7.87×10^−12^). Notably, this gene list includes *Gp1bb* (fold-change of −2.203 in *Pik3cg^−/−^* compared to wild type mice), *Gp5* (−3.211), *Gp9* (−0.996), *Gp6* (−0.711) and *Vwf* (−1.381). All five genes are transcribed in the megakaryocytic lineage in humans, based on the HaemAtlas data. The platelet glycoproteins GP1BB, GP5 and GP9, together with GP1BA constitute the platelet membrane receptor for the plasma protein Von Willebrand Factor (VWF), which is encoded by *VWF* [21].

### Canonical pathway enrichment analysis and protein–protein interaction network

To explore the signaling pathways of PIK3CG in humans, we analyzed 191 human orthologs of the 220 differentially expressed genes between *Pik3cg^−/−^* and wild type mice. Canonical pathway enrichment analysis based on the curated gene sets of the Molecular Signatures Database (MSigDB) v3.6 [22] revealed that the top six enriched gene sets were related to platelets: ‘platelet degranulation’ (*P*=3.44×10^−8^), ‘platelet activation’ (*P*=5.05×10^−8^), ‘formation of platelet plug’ (*P*=2.85×10^−7^), ‘hemostasis’ (*P*=7.48×10^−6^), ‘formation of fibrin clot and clotting cascade’ (*P*=1.39×10^−5^) and ‘platelet adhesion to exposed collagen’ (*P*=1.86×10^−5^).

**Figure 4:**
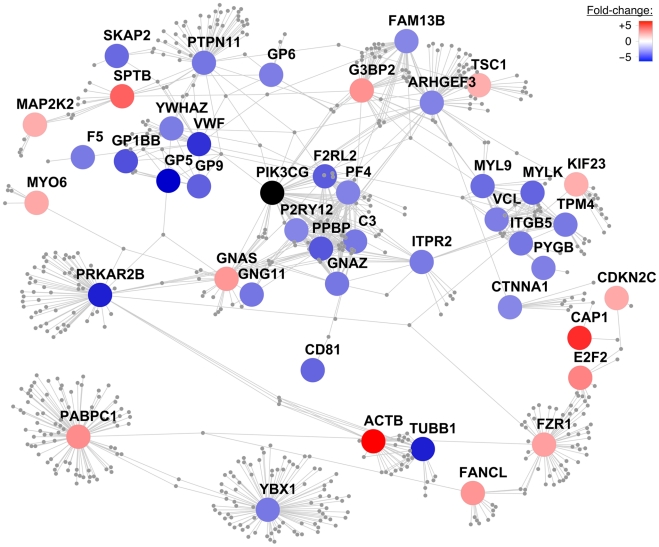
Protein–protein interaction network centered on PIK3CG. A protein-protein interaction network was constructed centering on the human orthologous proteins encoded by the 220 differentially expressed transcripts between *Pik3cg* knockout and wild type mice with a fold-change of at least ±1.5. The color of these ‘core’ proteins represents the fold-change of gene transcript levels between *Pik3cg^−/−^* and wild type mice on a continuous scale from over- (red) to underexpression (blue) of transcripts in knockout mice. Core proteins, which do not exhibit first-order interactors in the Reactome database and are disconnected from the largest connected component, are not shown. First-order interactors of PIK3CG that form network nodes and edges are shown in grey and were obtained from Reactome. Interactors that are not expressed in MK cells based on the HaemAtlas data are omitted. The resulting network consists of 45 core proteins with 642 nodes and 1067 edges.

We constructed a protein-protein interaction network centered on the proteins encoded by the 191 transcripts described above. First-order interactors of these ‘core’ proteins were obtained from Reactome, an open-source manually curated database of human biological pathways [23], [24]. We filtered interactors on their expression levels in MK cells (Materials and Methods). The resulting network incorporated 45 core proteins centered on PIK3CG consisting of 642 nodes and 1067 edges (Figure 4).

## Discussion

The intersection of maps of open chromatin with variants identified through GWA studies can facilitate the search for underlying functional variant(s). We applied the FAIRE assay to generate a catalog of NDRs in a megakaryocytic and an erythroblastoid cell line at 62 selected genetic loci associated with hematological and cardiovascular traits. We provided evidence that open chromatin profiles exhibit distinct patterns among different cell types and that cell-specific NDRs can be useful in prioritizing regions for further functional analysis. As proof, we elucidated the molecular basis of the association with mean platelet volume and function at chromosome 7q22.3. We identified a NDR in MK but not EB cells containing the index SNP rs342293 for this association and demonstrated that the alleles of rs342293 differentially bind the transcription factor EVI1. Thus, to the best of our knowledge this is the first functional variant to be elucidated among the known platelet QTLs.

Expression QTL data in platelets and macrophages provided statistical support that rs342293 affects *PIK3CG* gene expression levels. *PIK3CG* is transcribed in megakaryocytes but only weakly expressed in erythroblasts [18], which is in agreement with the MK-specific properties of the identified NDR. However, additional work is required to scrutinize all possible targets of this regulatory module. The closest gene *FLJ36031*, which spans only 2 kb, has no reported protein product and function and requires further characterization. *PIK3CG* is located 134 kb downstream of rs342293 and encodes the phosphoinositide-3-kinase γ-catalytic subunit. The lipid kinase PIK3CG (PI3Kγ) is a member of the class I PI3Ks and catalyzes the conversion of phosphatidylinositol-4,5-bisphosphate (PtdIns(4,5)P2; PIP2) to phosphatidylinositol-3,4,5-trisphosphate (PtdIns(3,4,5)P3; PIP3) downstream of cell surface receptor activation [25]–[27]. In megakaryocytes and platelets, PIP3 is crucial in the collagen-induced regulation of phospholipase C and initiation of megakaryopoiesis and proplatelet formation [28]. Functional studies with *Pik3cg* knockout mice have indicated a role in wound healing [29], ADP-induced platelet aggregation and thrombosis [30], [31]. It is noteworthy that PIK3CG has also a prominent role in macrophage activation [27].

Whole-genome gene expression profiling in *Pik3cg* ^−/−^ mice showed differential expression of genes involved in important platelet-related pathways compared to wild type mice, most notably *Vwf* and its platelet membrane receptor components. Our recent meta-analysis of GWA studies in 68,000 Northern Europeans showed that common sequence variation at the *VWF* and *GP1BA* loci exert an effect on platelet volume and counts, respectively (NS, unpublished data). This recent finding together with the established knowledge that Mendelian mutations in the genes encoding the platelet VWF (Von Willebrand disease, type 2; OMIM: 613554) and its receptor (Bernard-Soulier syndrome; OMIM: 231200) are causative of giant platelets, give biological credence to the observed effects of *Pik3cg* knockout on the transcription of these platelet genes. The protein-protein interaction network centered on PIK3CG highlighted additional proteins implicated in severe platelet disorders, including TUBB1 (macrothrombocytopenia, autosomal dominant, TUBB1-related; OMIM: 613112), F5 (factor V deficiency; OMIM: 612309) and P2RY12 (bleeding disorder due to P2RY12 defect; OMIM: 609821). Therefore, it is plausible to assume that differences in the *PIK3CG* transcript levels in humans, based on the different alleles of rs342293, may lead to changes in the abundance of platelet membrane proteins that are key regulators of platelet formation.

We previously showed an association of rs342293-G with decreased platelet reactivity in humans, assessed as the proportion of binding to annexin V and fibrinogen, as well as P-selectin expression, after activation of platelets with collagen-related peptide (CRP-XL) [20]. We also observed that rs342293 is likely to modify events downstream of signaling via the collagen signaling receptor glycoprotein VI, which is encoded by *GP6* [32]. A recent GWA study showed the same locus at chromosome 7q22.3 to be associated also with epinephrine-induced platelet aggregation [33]. The index SNP in that study, rs342286, and rs342293 are in high LD (r^2^=0.87, Phase II HapMap, CEU) making rs342293 the putative causative variant underlying both functional associations. The observation that platelet functional events triggered via an immunoreceptor tyrosine-based activation motif on the Fc receptor γ-chain (ITAM-FcR-γ) or G-protein-coupled receptor, for collagen and epinephrine, respectively, are both modified by differences in the *PIK3CG* transcript level is compatible with the notion that both signaling cascades require PIP3 and that silencing of *Pik3cg* in mice reduces the expression of the *Gp6* gene.

**Figure 5:**
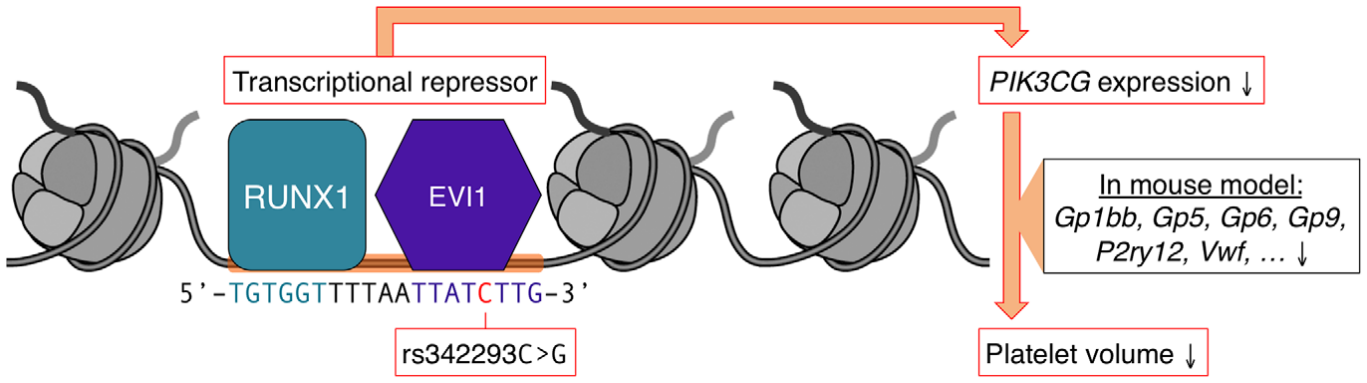
Model of the mechanism by which rs342293 may affect platelet volume. Based on our data, we propose that rs342293C>G affects EVI1 transcription factor binding at the megakaryocyte-specific site of open chromatin at chromosome 7q22.3. The underlying DNA sequence around rs342293 (indicated in red) is shown and transcription factor binding motifs for RUNX1 (green) and EVI1 (purple) are highlighted. The transcriptional complex of EVI1 is a well-described repressor of gene transcription. In platelets, individuals with homozygous rs342293-C have lower *PIK3CG* gene expression levels compared to subjects with homozygous rs342293-G. In *Pik3cg* knockout mouse models, transcripts that encode key proteins for platelet membrane biogenesis were found to be downregulated, which may ultimately affect platelet phenotype.

Based on the above data, we propose a model (Figure 5) in which the DNA sequence containing the C- but not the G-allele of rs342293 binds the transcription factor EVI1, which acts as transcriptional repressor of *PIK3CG* in megakaryocytes and ultimately affects platelet phenotype.

*EVI1* (ecotropic viral integration site-1) encodes a protein with two zinc-finger domains (ZF1 and ZF2), which feature distinct DNA-binding specificities [34]. EVI1 mainly promotes hematopoietic differentiation into the megakaryocytic lineage. EVI1 was frequently reported to be a repressor of transcription that has the potential to recruit diverse regulatory proteins. For example, EVI1 antagonizes the growth-inhibitory effect of transforming growth factor-β (TGF-β), a potent regulator of megakaryopoiesis [35], by interacting with SMAD3 via ZF1, and inhibiting SMAD3 from binding to DNA [36], [37]. EVI1 contains domains that interact with RUNX1 (Runt-related transcription factor 1), which is the α-subunit of the transcription factor CBF (core binding factor). The interaction of EVI1 with the DNA-binding domain Runt of *RUNX1* leads to the destabilization of the DNA-RUNX1 complex and subsequent loss of RUNX1 function [38].

By converting the read-out system from microarrays to high-throughput next-generation sequencing, genome-wide open chromatin profiles can be interrogated. This would scale up analysis to the whole genome generating a catalog of open chromatin profiles to annotate association loci identified in past and future GWA studies. Furthermore, resources such as the completed 1000 Genomes Project will make the requirement for resequencing any identified NDR redundant. However, access to cell types relevant to the studied trait is not always feasible and can be a limitation for functional studies. For instance, we did not observe any intersection of FAIRE peaks with variants associated with CAD/MI or hypertension indicating that cell types other than the megakaryocytic and erythroblastoid cell lines analyzed here may be more suitable.

In addition to rs342293, we identified six putative functional variants associated with hematological and cardiovascular traits that fall into open chromatin (Table 2). Follow-up functional studies of these variants will further test the applicability of the approach we have proposed here. For example, the MK-specific NDR containing rs2038479 was found to mark an alternative promoter of a dynamin 3 (*DNM3*) transcript with expression restricted to the megakaryocytic lineage (WHO, unpublished data). Correlation of signatures of open chromatin with experimentally determined transcription factor binding sites studied in different cell types can systematically and rapidly translate GWA signals into functional components and biological mechanisms, thus paving the way to clinical benefit.

## Materials and Methods

### Ethics statement

All human subjects were recruited with appropriate informed consent in Cambridgeshire and enrolled in the Cambridge BioResource (http://www.cambridgebioresource.org.uk/). The study has ethical approval from the NHS Cambridgeshire Research Ethics Committee. The care and use of all mice in this study was carried out in accordance with the UK Home Office regulations under the Animals (Scientific Procedures) Act 1986.

### DNA tiling microarray design

We designed a 385,000-oligonucleotide tiling array (Roche NimbleGen) using 72 known genetic loci associated with the traits listed in Table 1 (National Human Genome Research Institute catalog of published GWA studies, http://www.genome.gov/gwastudies/). We considered only genetic loci that reached genome-wide significance with the threshold of *P*<5×10^−8^ (or as otherwise indicated in Table S9A) in a GWA study conducted with individuals of Northern and Western European ancestry (CEU population). In addition, we selected genetic loci based on biological evidence, where there was suggestive evidence of association. For each locus, we included the entire genetic region of the index SNP as defined by recombination hotspots based on Phase II HapMap [39]. If a recombination interval exceeded 500 kb, we included the closest target gene ±10 kb. In order to assess patterns of cell type specificity, we included eight lineage-specific reference genes for each of megakaryocytes, erythroblasts and monocytes on the array (Table S9B). We selected these transcripts on the basis of their expression profiles (Figure S5D) [18]. The oligonucleotide (50–75-mer probes) tiling array provided a mean probe span of 23 bp and harbored only probes unique to the human genome (build: hg18, coverage: 79%). In summary, a total of 62 unique complex trait loci and 24 reference gene loci representing 9.59 Mb and 1.77 Mb of genomic DNA, respectively, were selected for the array design.

### Formaldehyde-assisted isolation of regulatory elements (FAIRE)

We performed FAIRE in the human cell lines CHRF-288-11 and K562 on tiling arrays. Both cell lines exhibit markers characteristic for megakaryocytes/platelets [40], [41] and erythroblasts [42], [43], respectively. CHRF-288-11 cells were maintained in RPMI-1640 medium, supplemented with 20% horse serum (heat-inactivated) and 1% L-glutamine-penicillin-streptomycin solution. K562 cells were maintained in RPMI-1640 medium, supplemented with 10% fetal bovine serum (non-heat-inactivated), 2 mM GlutaMAX-I and 1% L-glutamine-penicillin-streptomycin solution. Cells were grown at 37°C, 5% CO_2_ and 100% humidified atmosphere. FAIRE experiments were carried out as previously described [44]. Briefly, for each cell type, 20×10^6^ cells were cross-linked with 1% formaldehyde for 8 or 12 min. Chromatin was extracted and subjected to 12 sonication cycles (30 sec of high pulse, 30 sec of rest) using the Bioruptor UCD-200 (Diagenode). DNA fragments depleted of proteins (‘open chromatin’) were phenol-chloroform extracted and ethanol precipitated. The samples were treated with RNase A and cleaned-up using the MinElute PCR Purification Kit (QIAGEN). A DNA amplification step was not applied.

### Detection of FAIRE DNA

DNA extracted from cross-linked cells (FAIRE, input sample) and uncross-linked cells (reference sample) were labeled with Cy5 and Cy3 dye, respectively, and subsequently co-hybridized to the tiling array. Dual-color sample labeling, array hybridization, washing and scanning were performed using the dual-channel microarray platform by Roche NimbleGen according to the manufacturer's protocol (NimbleGen Arrays User's Guide, ChIP-chip Analysis v4.1).

### FAIRE data analysis

Experimental data were analyzed using the software NimbleScan v2.5 (Roche NimbleGen). The two-channel raw signal intensities were scaled between channels by subtracting the Tukey bi-weight mean for the log_2_-ratio values for all features from each log_2_-ratio value. In the scaled log_2_-ratio data, peaks were identified using a sliding window approach [45]. For each chromosome, the log_2_-ratio cut-off value was calculated as the percentage of a hypothetical maximum (*P*_max_=arithmetic mean+6× standard deviation). The peak finding process was repeated using a series of log_2_-ratio cut-off values from *P*_start_ to *P*_end_. The following settings for the peak-finding analysis were applied: sliding window: 300 bp; min. probes>cut-off in peak=4; all probes in peak>cut-off=2; *P*_start_=90%, *P*_end_=15%, *P*_step_=0.5, number of steps: 100. Log_2_-ratio and peak data sets were displayed as UCSC Genome Browser (http://genome.ucsc.edu/) custom tracks. For the genomic characterization of FAIRE peaks, we extracted all annotation from the Ensembl database v54 (build: hg18). Proxy-SNPs to reported GWA index SNPs were gathered using the Genome-wide Linkage Disequilibrium Repository and Search Engine (GLIDERS) [46], with the following settings: Phase II HapMap v23 (CEU); MAF limit≥0.05; r^2^ limit≥0.8; no distance limits. The FAIRE microarray data sets are available online in the Gene Expression Omnibus (GEO) database (http://www.ncbi.nlm.nih.gov/geo/) under accession number GSE25716.

### Capillary DNA sequencing

DNA samples from a total of 643 individuals of Northern European ancestry were subjected to capillary DNA sequencing of the targeted locus at chromosome 7q22.3. Details of the sequencing protocol are described online (http://www.sanger.ac.uk/resources/downloads/human/exoseq.html). DNA was amplified by PCR and applied to bi-directional sequencing using Big Dye chemistry on 3730 DNA sequencers (Applied Biosystems). We used the following primer pairs (for two sequence-tagged sites): 5′-TGG AAA ATT ACA AAA GTC CCA AA, 5′-GAG AAA GGA TCA TGA GGG AGA A; and 5′-ACA AAA GTC CCA AAA TTT CAC A, 5′-GAG AAA GGA TCA TGA GGG AGA. The resulting products map to the following genomic location: chr7:106,159,328–106,159,998 (671 bp) and chr7:106,159,337–106,159,998 (662 bp). Pre-processed sequence traces were analyzed using the semi-automated analysis software ExoTrace, developed at the Wellcome Trust Sanger Institute (http://www.sanger.ac.uk/resources/downloads/human/exoseq.html). Potential SNP positions were indicated by the software and then reviewed manually.

### Transcription factor binding site analysis

*In silico* transcription factor binding sites were predicted using the software MatInspector v8.01 [47], with the following parameters: matrix group: vertebrates; core=1.00; matrix=optimized+0.02; tissue: hematopoietic system.

### Electrophoretic mobility shift assay (EMSA)

Nuclear extract was prepared from CHRF-288-11 cells with the NE-PER Nuclear and Cytoplasmic Extraction Reagents (Thermo Fisher Scientific). Oligonucleotides were designed based on the genomic sequence surrounding rs342293; the SNP position is shown in bold: 5′-biotin-AGC CCT GTG GTT TTA ATT AT**C/G** TTG AGG TTC AGG CTC A. Competitor probes were prepared without biotin tags. The labeled strands were annealed with the unlabeled complementary strands using a standard protocol. All oligonucleotides were provided by Sigma-Aldrich. We performed gel mobility shift assays with the LightShift Chemiluminescent EMSA Kit (Thermo Fisher Scientific) according to the manufacturer's protocol. Each 20-µl binding reaction contained 1× binding buffer, 75 ng/µl poly(dI/dC), 2.5% glycerol, 0.05% NP-40, 87.5 mM KCl and 6.25 mM MgCl_2_. For competition assays, we used 200-fold molar excess of the unlabeled probe. Reactions were incubated for 2 hr at room temperature. Supershift experiments were performed with EVI1 (sc-8707 X, Santa Cruz Biotechnology), GATA1 (ab28839, Abcam) and RUNX1 (sc-28679 X, Santa Cruz Biotechnology) antibodies.

### Expression QTL analysis

Gene expression profiling and genotypic data sets were obtained from different sources depending on the studied cell type. RNA from all studied cell types was isolated using a TRIZOL standard protocol. Total RNA was quantified using the NanoDrop (Labtech International) and quality-checked using the 2100 Bioanalyzer (Agilent Technologies). Standard protocols were applied to generate biotinylated cRNA and hybridize to Illumina BeadChips (platelets: HumanWG-6 v2; macrophages/monocytes: HumanRef-8 v3; LCLs/fat/skin: HumanHT-12 v3). Then, arrays were washed and scanned. All experimental procedures were carried out according to the manufacturer's protocol. Even though different versions of Illumina platforms were used, the probe for *PIK3CG* is the same across all chips (probe-ID: ILMN_1770433). Genotyping was performed on DNA extracted from whole blood using TaqMan (Applied Biosystems) SNP genotyping assays (platelets study) and Illumina SNP arrays (macrophages/monocytes study: Human 1.2M-Custom and Human 670-Quad-Custom; LCLs/fat/skin study: Human 1M-Duo) following the manufacturer's instructions. Expression QTL analysis with rs342293 or its proxy-SNP was performed with the software Genevar (Gene Expression Variation) using a window of ±1 Mb centered on the SNP [48]. The strength of the relationship between alleles and gene expression intensities was estimated using Spearman's rank correlation and reported as nominal *P*-values.

### Whole-genome gene expression profiling of *Pik3cg^−/−^* mice

*Pik3cg* knockout mice were obtained from sources previously described [49], backcrossed onto the C57BL/6J Jax genetic background for eight generations (B6J;129-Pik3cg*^tm1Pngr^*) and then maintained as a closed colony by intercrossing from within the colony (C57BL/6J Jax contribution: 99.6%). We performed PCR genotyping using a standard protocol with the following primer pairs: 5′-TCA GGC TCG GAG ATT AGG TA, 5′-GCC CAA TCG GTG GTA GAA CT (wild type); 5′-GGA CAC GGC TTT GAT TAC AAT C, 5′-GGG GTG GGA TTA GAT AAA TG (mutant) [49]. Whole blood from three *Pik3cg^−/−^* and three C57BL/6J Jax wild type mice (all females, age: 13–16 weeks, Mouse Breeders Diet (Lab Diets 5021-3)) was collected from terminally anesthetized mice via the retro-orbital sinus. We extracted total RNA using the Mouse RiboPure-Blood RNA Isolation Kit (Ambion). Total RNA was quantified using the NanoDrop and quality-checked using the 2100 Bioanalyzer. Standard protocols were used to generate biotinylated cRNA and hybridize to MouseWG-6 v2 Expression BeadChips. Arrays were washed, detected and scanned. All experimental procedures were carried out according to the manufacturer's protocol. On the raw expression data, we performed background subtraction, variance-stabilizing transformation and quantile normalization across all samples with the R package lumi (http://bioconductor.org/packages/release/bioc/html/lumi.html). Technical replicates were averaged and the differentially expressed transcripts between wild type and knockout mice were identified by calculating the log_2_-fold changes of the averaged expression values. *P*-values were calculated by 1-way analysis of variance (ANOVA). We performed gene ontology term enrichment analysis using AmiGO v1.7 [50], with the following parameters: gene expression fold-change cut-off: ±1.5; background: MGI; *P*-value cut-off: 1×10^−5^; minimum number of gene products: 10. The whole-genome gene expression data sets of *Pik3cg^−/−^* and wild type mice are available online in the GEO database under accession number GSE26111.

### Protein–protein interaction network

Of the 220 differentially expressed genes between *Pik3cg^−/−^* and wild type mice, we retrieved 191 orthologous human genes using BioMart (http://www.ensembl.org/biomart/martview/) and their respective proteins using UniProt (http://www.uniprot.org/). These ‘core’ proteins were used as primary seeds to develop the protein-protein interaction network. We determined first-order interactors of core proteins using Reactome v36. Only clustered non-redundant first-level interactions between human proteins that were connected to the largest connected component were considered. Based on the HaemAtlas data, we neglected interactors that are not expressed in MK cells (*P*>0.01). The described network is available for download in Cytoscape v2.8.0 format (Dataset S1).

## Supporting Information

Figure S1 Analytical approach for peak calling in FAIRE-chip data sets. FAIRE was performed with different formaldehyde cross-linking times (8 and 12 min). In order to reduce experimental error and achieve higher stringency, replicated and overlapping peaks were merged for each cell type and subjected to further analysis.
(TIF)

Figure S2 Location of the open chromatin sites in respect to the closest transcription start site (TSS) at the selected association loci.
(TIF)

Figure S3 Average number of FAIRE peaks in lineage-specific genes in MK and EB cells. The number of open chromatin sites in lineage-specific genes (±2 kb) was averaged and normalized for the length of the gene.
(TIF)

Figure S4 Maps of open chromatin at the selected genetic loci in MK and EB cells displayed as UCSC Genome Browser custom tracks. (A) *FLJ36031–PIK3CG*; (B) *DNM3*; (C) *HBS1L–MYB*; (D) *PEAR1*; (E) *RAF1*; (F) *TMCC2*; (G) *CYP17A1–C10orf32*. Shown are the scaled log_2_-ratio and the called peaks from FAIRE experiments in an erythroblastoid (blue) and a megakaryocytic cell line (red). Only the data sets using a formaldehyde fixation time of 12 min are shown for both cell types. The putative regulatory SNP located within a site of open chromatin is shown below each track.
(PDF)

Figure S5 Gene expression profiles. The heat maps show normalized gene expression profiles in differentiated human blood cells according to the HaemAtlas (Materials and Methods). (A) Gene loci that harbor cell type-specific open chromatin and putative regulatory sequence variants; (B) Transcription factors predicted to bind DNA sequence motifs around rs342293; (C) Genes within a 1-Mb interval of rs342293; (D) Lineage-specific reference genes.
(TIF)

Figure S6 Gel shift assays in CHRF-288-11 nuclear extracts using GATA1 and RUNX1 antibodies. No supershift was observed when incubating CHRF-288-11 nuclear extract with GATA1 antibodies for probes containing either rs342293-C or -G. However, we showed evidence for RUNX1 transcription factor binding *in vitro*. Reactions were incubated for 1 hr at room temperature.
(TIF)

Figure S7 No significant GATA1 but weak RUNX1 binding at the MK-specific open chromatin region at chromosome 7q22.3. Cord blood-derived CD34-positive hematopoietic progenitor cells were seeded at 1×10^5^ in CellGro SCGM (CellGenix) in the presence of 100 ng/ml recombinant human thrombopoietin (CellGenix) and 10 ng/ml interleukin-1β (Miltenyi Biotec) for 10 days, after which 71% of cells expressed CD41. ChIP was performed as previously described [51] with GATA1 (ab11963, Abcam) and RUNX1 (ab23980, Abcam) antibodies. Samples were amplified and sequenced on the Illumina Genome Analyzer II following manufacturer's instructions. Data were transformed into density plots and displayed as UCSC Genome Browser custom tracks. Visual inspection of the 7q22.3 region showed no *in vivo* binding of GATA1, but weak RUNX1 binding. In total, the ChIP-seq data sets comprised 4,722 and 7,345 peaks for GATA1 and RUNX1, respectively [52].
(TIF)

Table S1 FAIRE peak data sets. The genomic positions of FAIRE peaks are reported at (A) the association loci and (B) the lineage-specific reference genes.
(PDF)

Table S2 Comparison of the FAIRE peak density between the ENCODE and the here presented data sets.
(PDF)

Table S3 Characterization of open chromatin regions in relation to gene annotations.
(PDF)

Table S4 Investigation of the functional role of platelet volume-associated variants at chromosome 7q22.3.
(PDF)

Table S5 Resequencing of the MK-specific open chromatin region at chromosome 7q22.3.
(PDF)

Table S6 Expression QTL associations at the *PIK3CG* gene locus in platelets, macrophages, monocytes, B cells (LCLs), fat, and skin.
(PDF)

Table S7 Differentially expressed genes between *Pik3cg^−/−^* and wild-type mice.
(PDF)

Table S8 Functional ontology classification of differentially expressed genes between *Pik3cg^−/−^* and wild-type mice.
(PDF)

Table S9 Genetic loci selected for the high-density DNA tiling array.
(PDF)

Dataset S1 PIK3CG protein–protein interaction network.
(BZ2)
