# Correction: Maps of Open Chromatin Guide the Functional Follow-Up of Genome-Wide Association Signals: Application to Hematological Traits

**DOI:** 10.1371/annotation/5d0c3be4-6f34-420a-960f-0a880bbf6128

**Published:** 2011-07-21

**Authors:** Dirk S. Paul, James P. Nisbet, Tsun-Po Yang, Stuart Meacham, Augusto Rendon, Katta Hautaviita, Jonna Tallila, Jacqui White, Marloes R. Tijssen, Suthesh Sivapalaratnam, Hanneke Basart, Mieke D. Trip, Consortium Cardiogenics, Consortium MuTHER, Berthold Göttgens, Nicole Soranzo, Willem H. Ouwehand, Panos Deloukas

The following information was omitted from the financial disclosure:

'MRT was supported by the Netherlands Organisation for Scientific Research (NWO; 2008/05927/ALW).'

