# Supplementary figures and images for: Maps of Open Chromatin Guide the Functional Follow-Up of Genome-Wide Association Signals: Application to Hematological Traits
Source: PLoS Genet. 2011 Jun 30;7(6):e1002139. doi: 10.1371/journal.pgen.1002139 (PMC3128100; doi:10.1371/journal.pgen.1002139)

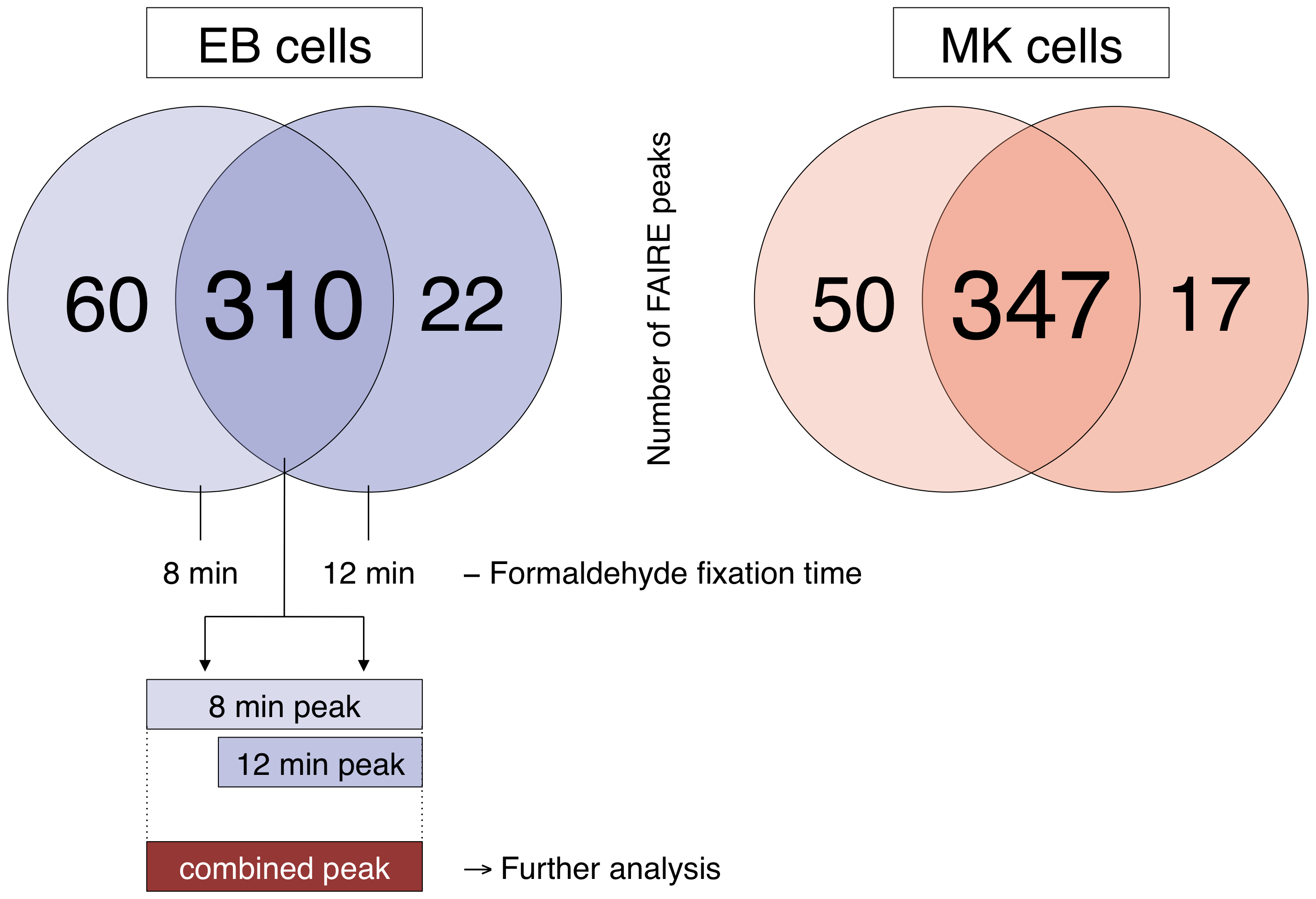

Supplement: Figure S1 — Analytical approach for peak calling in FAIRE-chip data sets. FAIRE was performed with different formaldehyde cross-linking times (8 and 12 min). In order to reduce experimental error and achieve higher stringency, replicated and overlapping peaks were merged for each cell type and subjected to further analysis. (TIF) [file pgen.1002139.s001.tif]

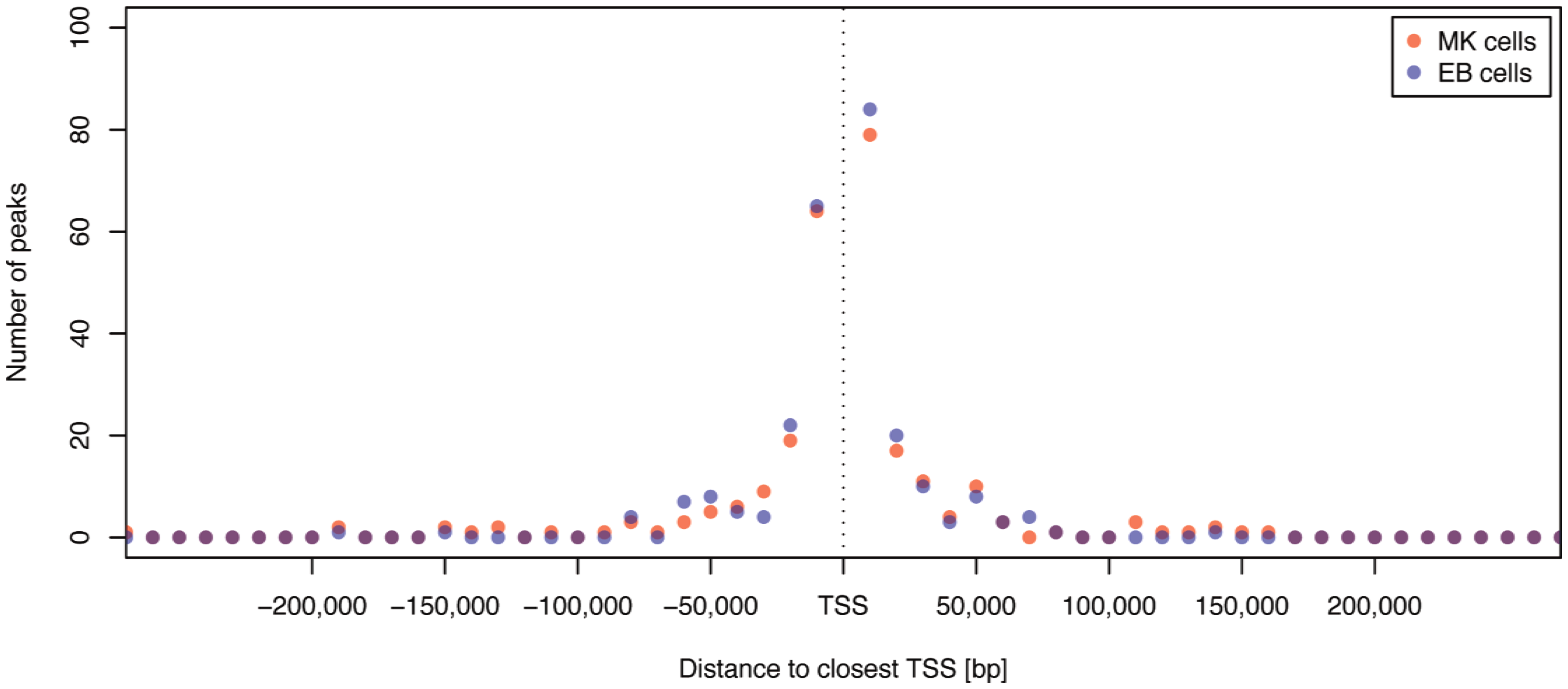

Supplement: Figure S2 — Location of the open chromatin sites in respect to the closest transcription start site (TSS) at the selected association loci. (TIF) [file pgen.1002139.s002.tif]

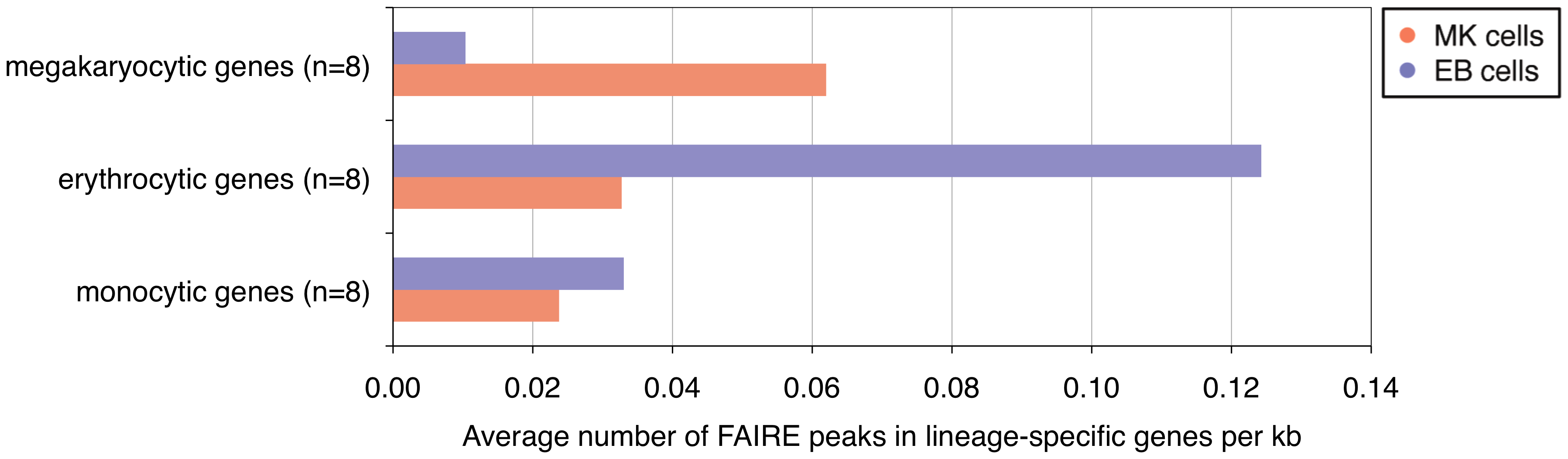

Supplement: Figure S3 — Average number of FAIRE peaks in lineage-specific genes in MK and EB cells. The number of open chromatin sites in lineage-specific genes (±2 kb) was averaged and normalized for the length of the gene. (TIF) [file pgen.1002139.s003.tif]

(A) *FLJ36031–PIK3CG*

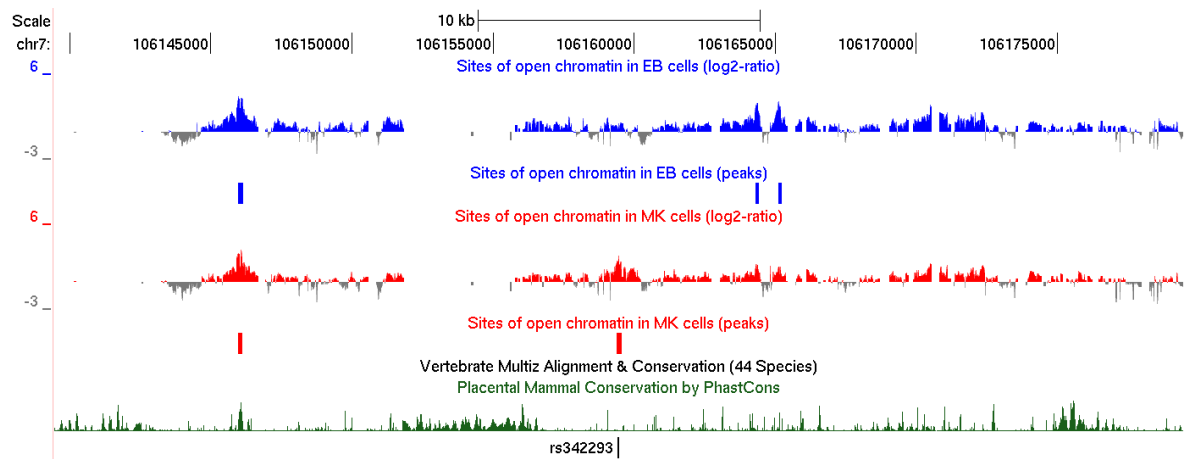

(B) *DNM3*

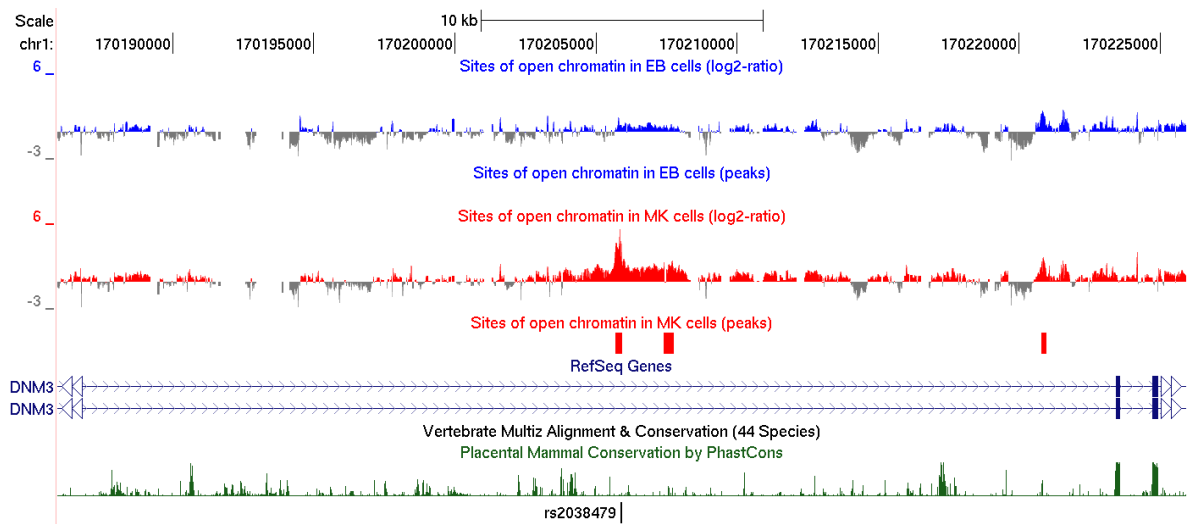

(C) *HBS1L–MYB*

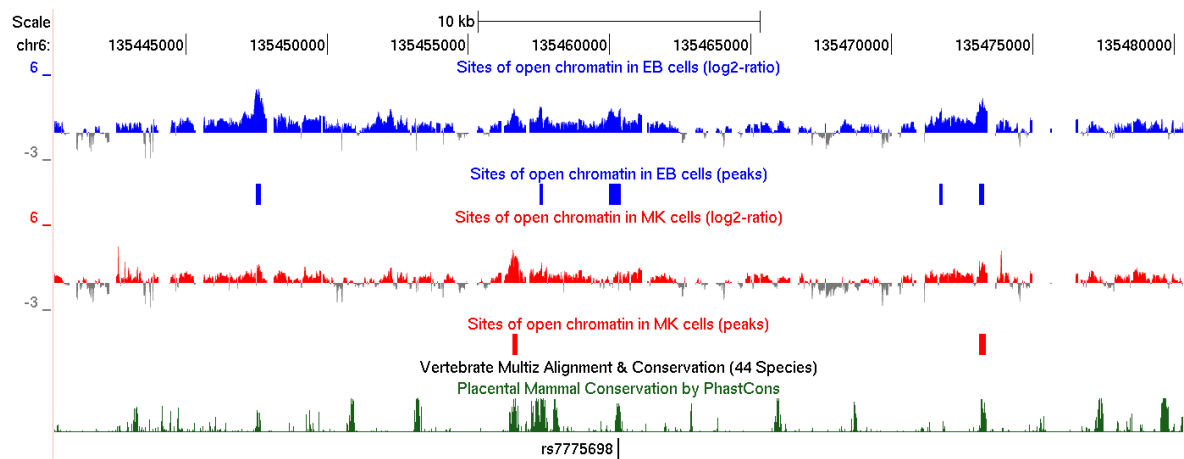

**(D) *PEAR1***

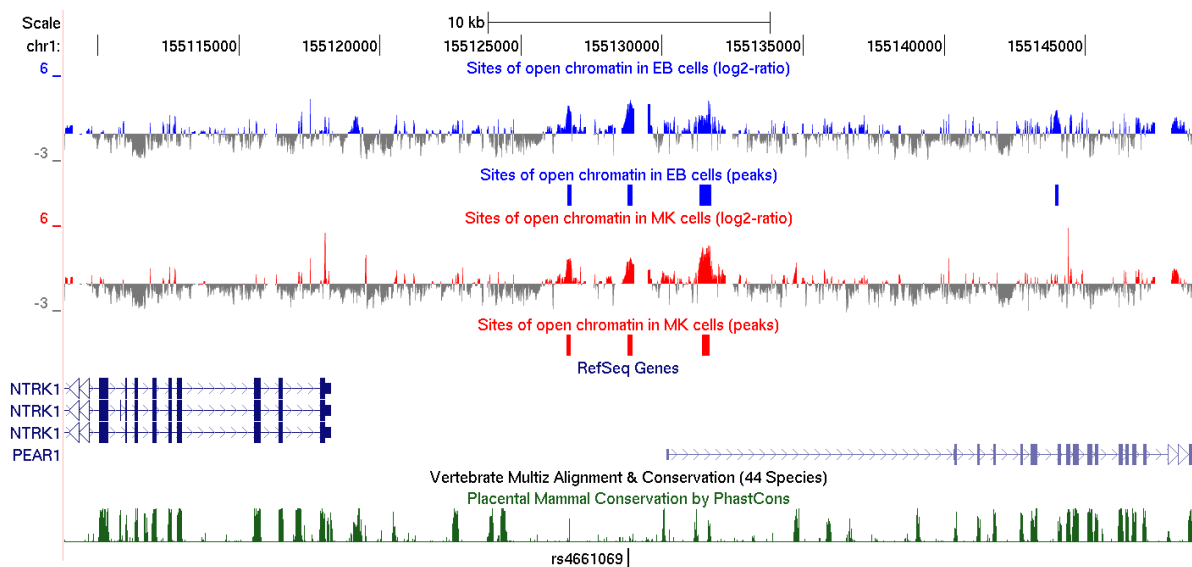

**(E) *RAF1***

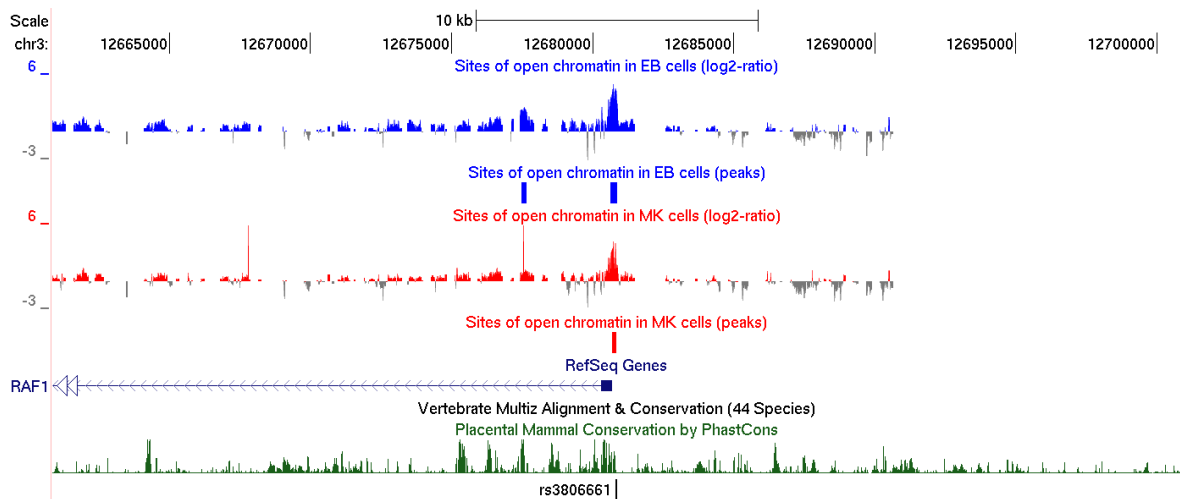

**(F) *TMCC2***

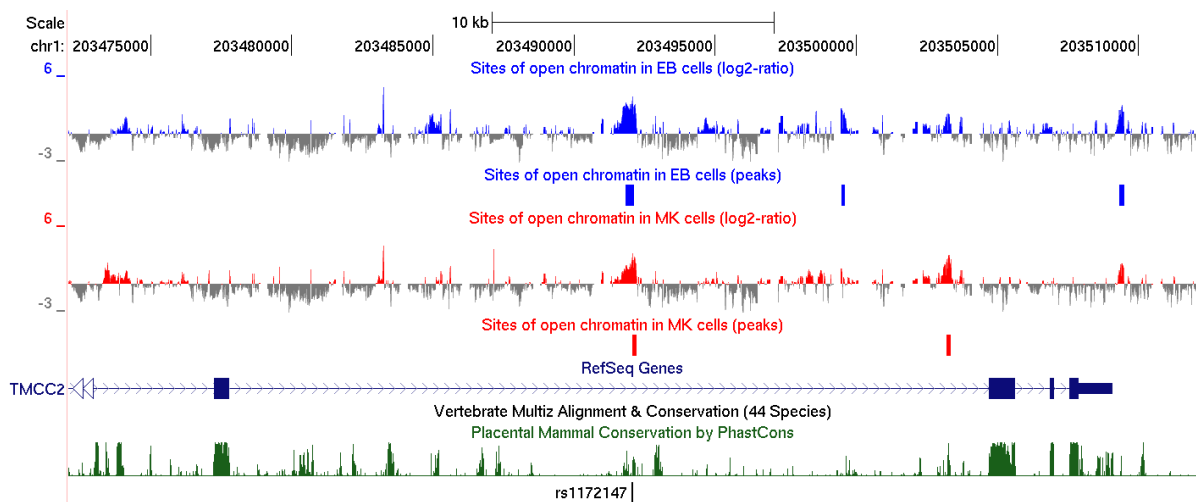

(G) *CYP17A1–C10orf32*

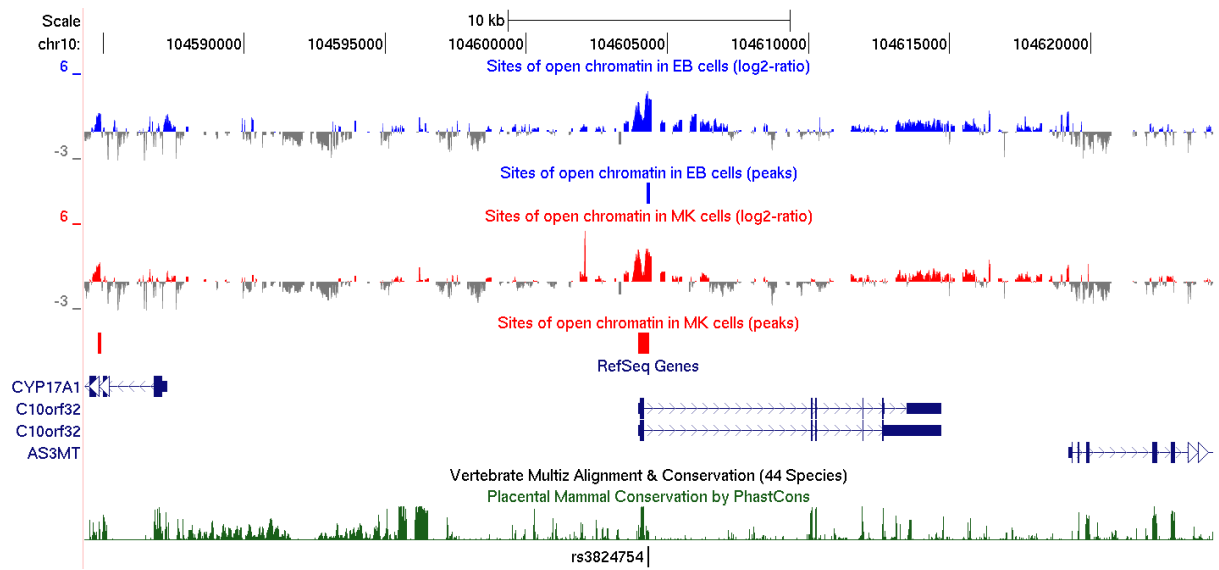

Supplement: Figure S4 — Maps of open chromatin at the selected genetic loci in MK and EB cells displayed as UCSC Genome Browser custom tracks. (A) FLJ36031–PIK3CG; (B) DNM3; (C) HBS1L–MYB; (D) PEAR1; (E) RAF1; (F) TMCC2; (G) CYP17A1–C10orf32. Shown are the scaled log2-ratio and the called peaks from FAIRE experiments in an erythroblastoid (blue) and a megakaryocytic cell line (red). Only the data sets using a formaldehyde fixation time of 12 min are shown for both cell types. The putative regulatory SNP located within a site of open chromatin is shown below each track. (PDF) [file pgen.1002139.s004.pdf]

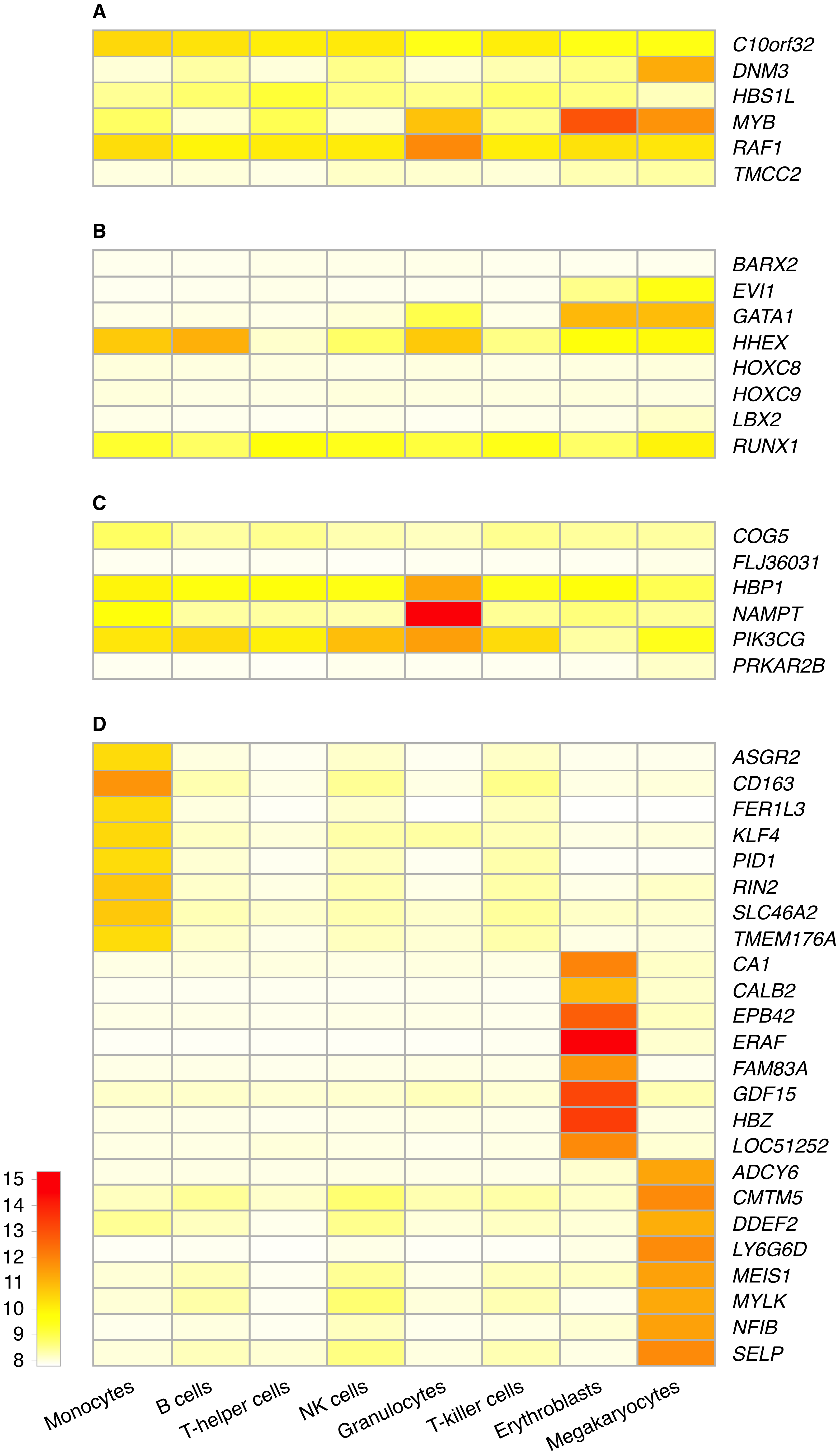

Supplement: Figure S5 — Gene expression profiles. The heat maps show normalized gene expression profiles in differentiated human blood cells according to the HaemAtlas (Materials and Methods). (A) Gene loci that harbor cell type-specific open chromatin and putative regulatory sequence variants; (B) Transcription factors predicted to bind DNA sequence motifs around rs342293; (C) Genes within a 1-Mb interval of rs342293; (D) Lineage-specific reference genes. (TIF) [file pgen.1002139.s005.tif]

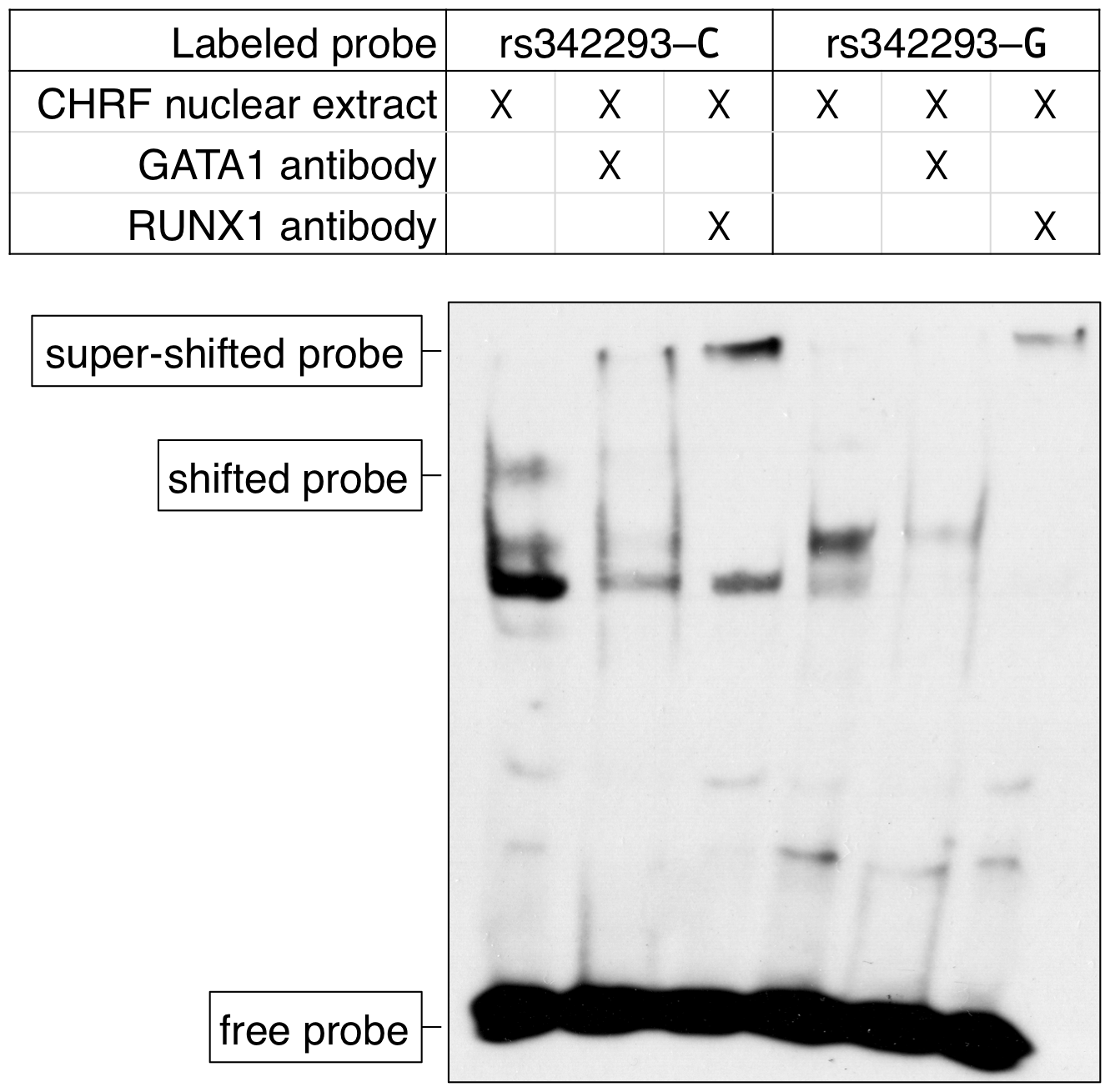

Supplement: Figure S6 — Gel shift assays in CHRF-288-11 nuclear extracts using GATA1 and RUNX1 antibodies. No supershift was observed when incubating CHRF-288-11 nuclear extract with GATA1 antibodies for probes containing either rs342293-C or -G. However, we showed evidence for RUNX1 transcription factor binding in vitro. Reactions were incubated for 1 hr at room temperature. (TIF) [file pgen.1002139.s006.tif]

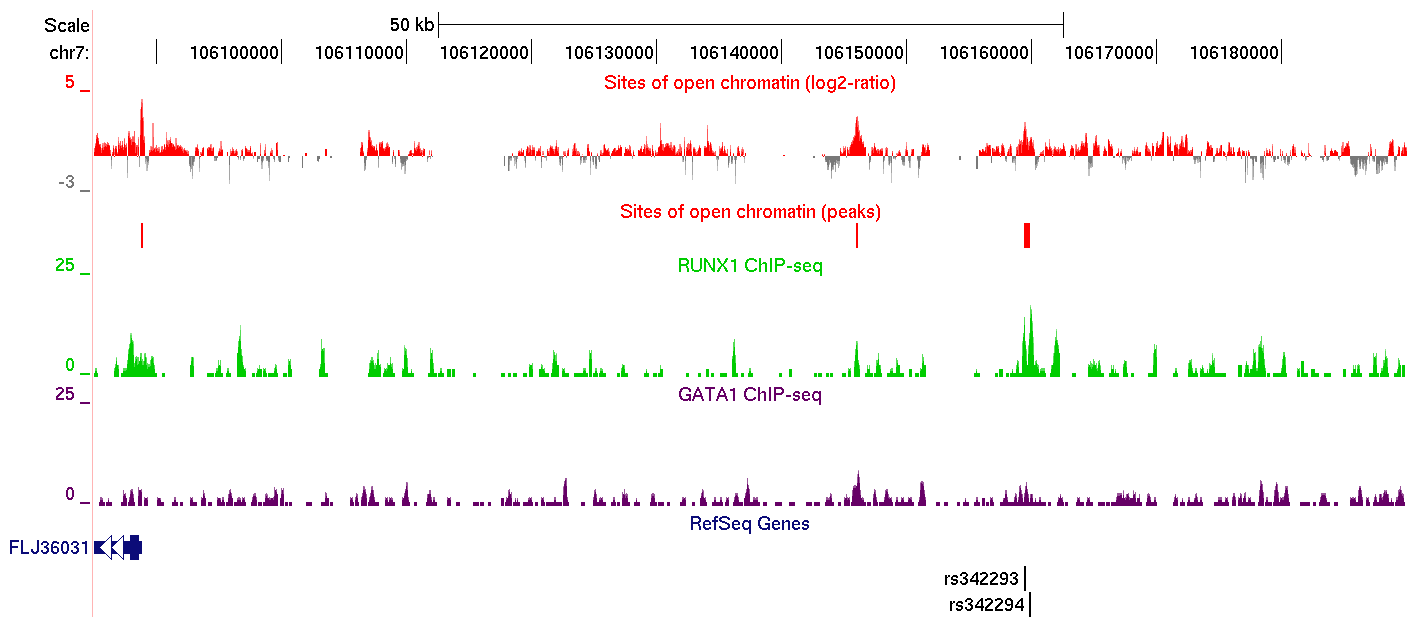

Supplement: Figure S7 — No significant GATA1 but weak RUNX1 binding at the MK-specific open chromatin region at chromosome 7q22.3. Cord blood-derived CD34-positive hematopoietic progenitor cells were seeded at 1×10^5^ in CellGro SCGM (CellGenix) in the presence of 100 ng/ml recombinant human thrombopoietin (CellGenix) and 10 ng/ml interleukin-1β (Miltenyi Biotec) for 10 days, after which 71% of cells expressed CD41. ChIP was performed as previously described [51] with GATA1 (ab11963, Abcam) and RUNX1 (ab23980, Abcam) antibodies. Samples were amplified and sequenced on the Illumina Genome Analyzer II following manufacturer's instructions. Data were transformed into density plots and displayed as UCSC Genome Browser custom tracks. Visual inspection of the 7q22.3 region showed no in vivo binding of GATA1, but weak RUNX1 binding. In total, the ChIP-seq data sets comprised 4,722 and 7,345 peaks for GATA1 and RUNX1, respectively [52]. (TIF) [file pgen.1002139.s007.tif]

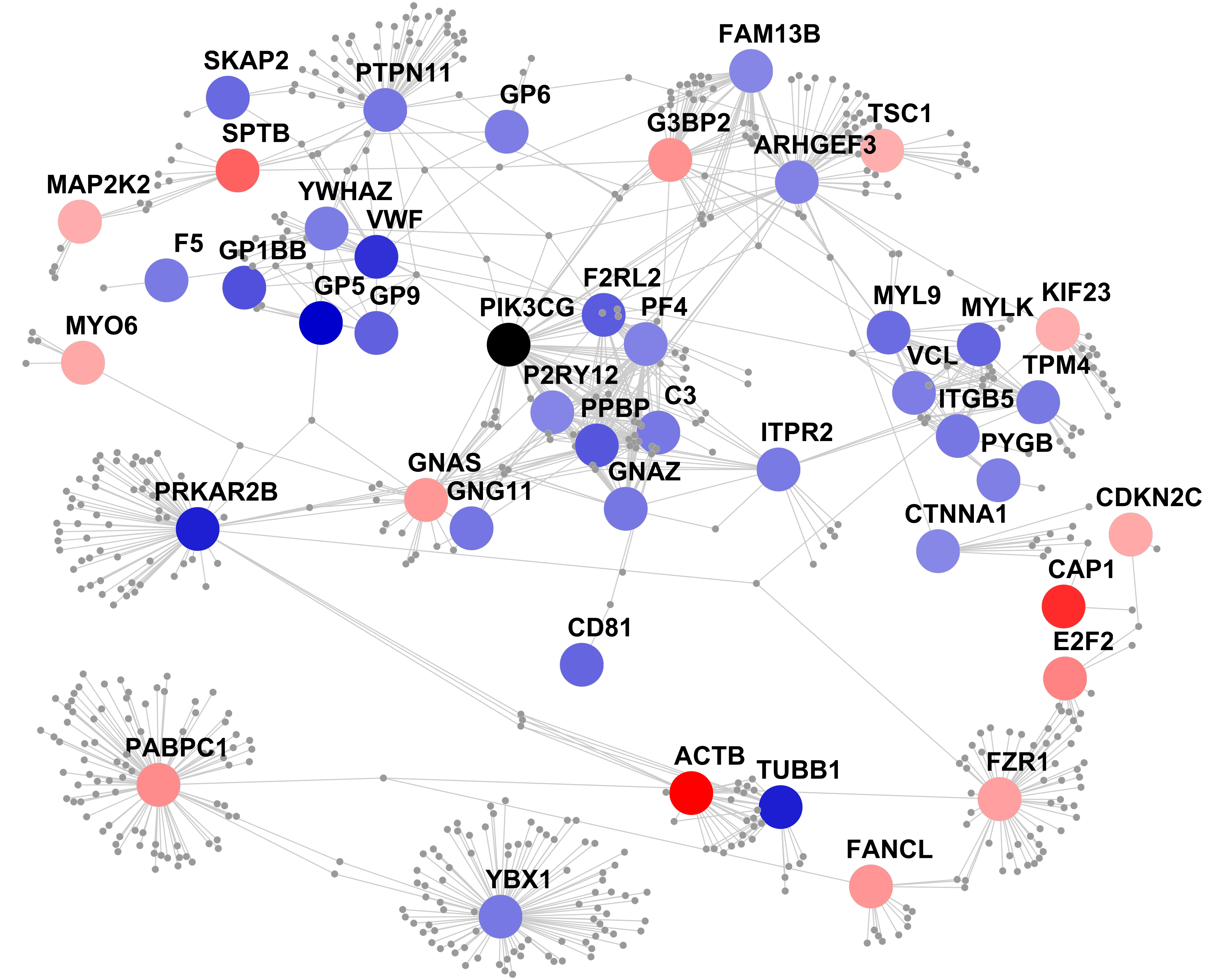

Supplement: Dataset S1 — PIK3CG protein–protein interaction network. (BZ2) [file pgen.1002139.s017.bz2 › Pik3cg_network/Pik3cg_network.png]
