# Supplementary material for: Maps of Open Chromatin Guide the Functional Follow-Up of Genome-Wide Association Signals: Application to Hematological Traits
Source: PLoS Genet. 2011 Jun 30;7(6):e1002139. doi: 10.1371/journal.pgen.1002139 (PMC3128100; doi:10.1371/journal.pgen.1002139)
Supplement: Table S1 — FAIRE peak data sets. The genomic positions of FAIRE peaks are reported at (A) the association loci and (B) the lineage-specific reference genes. (PDF) [file pgen.1002139.s008.pdf]

Table S1. FAIRE peak data sets.

(A) Association loci					
#	Cell type	Gene locus	Genomic position		
			Chromosome	Start	End
1	MK	*VAV3*	1	107,914,840	107,915,135
2	MK	*CELSR2, PSRC1, SORT1*	1	109,537,919	109,538,062
3	MK	*CELSR2, PSRC1, SORT1*	1	109,557,893	109,558,576
4	MK	*CELSR2, PSRC1, SORT1*	1	109,562,771	109,563,006
5	MK	*CELSR2, PSRC1, SORT1*	1	109,593,505	109,594,216
6	MK	*CELSR2, PSRC1, SORT1*	1	109,627,258	109,627,475
7	MK	*CELSR2, PSRC1, SORT1*	1	109,660,767	109,660,957
8	MK	*CELSR2, PSRC1, SORT1*	1	109,664,465	109,664,672
9	MK	*CELSR2, PSRC1, SORT1*	1	109,714,935	109,715,078
10	MK	*CELSR2, PSRC1, SORT1*	1	109,734,038	109,734,237
11	MK	*CELSR2, PSRC1, SORT1*	1	109,734,968	109,735,126
12	MK	*PEAR1*	1	155,102,735	155,102,938
13	MK	*PEAR1*	1	155,126,625	155,126,793
14	MK	*PEAR1*	1	155,128,772	155,128,965
15	MK	*PEAR1*	1	155,131,394	155,131,731
16	MK	*PEAR1*	1	155,194,762	155,194,983
17	MK	*FCER1G*	1	159,451,530	159,451,747
18	MK	*FCER1G*	1	159,453,843	159,454,060
19	MK	*FCER1G*	1	159,462,115	159,462,448
20	MK	*FCER1G*	1	159,463,886	159,464,219
21	MK	*DNM3*	1	170,184,382	170,184,657
22	MK	*DNM3*	1	170,205,693	170,205,939
23	MK	*DNM3*	1	170,207,408	170,207,753
24	MK	*DNM3*	1	170,220,764	170,220,954
25	MK	*DNM3*	1	170,238,612	170,238,785
26	MK	*DNM3*	1	170,239,997	170,240,212
27	MK	*DNM3*	1	170,246,245	170,246,374
28	MK	*DNM3*	1	170,345,175	170,345,373
29	MK	*TMCC2*	1	203,447,052	203,447,525
30	MK	*TMCC2*	1	203,463,544	203,463,663
31	MK	*TMCC2*	1	203,492,061	203,492,206
32	MK	*TMCC2*	1	203,503,192	203,503,337
33	MK	*TMCC2*	1	203,524,084	203,524,303
34	MK	*MIA3*	1	220,683,640	220,683,839
35	MK	*MIA3*	1	220,695,158	220,695,367
36	MK	*MIA3*	1	220,704,890	220,705,039
37	MK	*MIA3*	1	220,705,566	220,706,004
38	MK	*MIA3*	1	220,829,860	220,830,153
39	MK	*MIA3*	1	220,857,748	220,857,865
40	MK	*MIA3*	1	220,874,539	220,874,712

41	MK	*MIA3*	1	220,881,541	220,881,708
42	MK	*MIA3*	1	220,884,113	220,884,324
43	MK	*MIA3*	1	220,894,985	220,895,158
44	MK	*MIA3*	1	220,895,763	220,895,936
45	MK	*MIA3*	1	220,951,347	220,951,492
46	MK	*MIA3*	1	220,952,425	220,952,565
47	MK	*MIA3*	1	220,952,756	220,953,397
48	MK	*MIA3*	1	221,013,036	221,013,171
49	MK	*MIA3*	1	221,054,903	221,055,115
50	MK	*EHD3*	2	31,310,233	31,310,426
51	MK	*EHD3*	2	31,312,109	31,312,250
52	MK	*EHD3*	2	31,313,350	31,313,549
53	MK	*EHD3*	2	31,316,040	31,316,233
54	MK	*STK39*	2	168,701,192	168,701,417
55	MK	*WDR12*	2	203,444,209	203,444,704
56	MK	*WDR12*	2	203,484,606	203,484,932
57	MK	*WDR12*	2	203,485,121	203,485,242
58	MK	*ITPR1*	3	4,789,003	4,789,189
59	MK	*ITPR1*	3	4,823,523	4,823,700
60	MK	*ITPR1*	3	4,847,909	4,848,262
61	MK	*ITPR1*	3	4,851,141	4,851,312
62	MK	*ITPR1*	3	4,852,491	4,852,736
63	MK	*ITPR1*	3	4,853,989	4,854,492
64	MK	*RAF1*	3	12,680,681	12,680,868
65	MK	*ARHGEF3*	3	56,765,356	56,765,495
66	MK	*ARHGEF3*	3	56,793,061	56,793,222
67	MK	*ARHGEF3*	3	56,799,422	56,799,589
68	MK	*ARHGEF3*	3	56,808,646	56,808,811
69	MK	*ARHGEF3*	3	56,810,974	56,811,195
70	MK	*ARHGEF3*	3	56,824,732	56,824,917
71	MK	*ARHGEF3*	3	56,865,019	56,865,132
72	MK	*MRAS*	3	139,550,152	139,550,443
73	MK	*P2RY12*	3	152,428,299	152,428,498
74	MK	*P2RY12*	3	152,478,861	152,479,030
75	MK	*P2RY12*	3	152,483,531	152,483,685
76	MK	*P2RY12*	3	152,484,566	152,484,761
77	MK	*ITGA2*	5	52,280,195	52,280,370
78	MK	*ITGA2*	5	52,320,783	52,321,224
79	MK	*ITGA2*	5	52,384,112	52,384,247
80	MK	*ITGA2*	5	52,384,726	52,384,848
81	MK	*ITGA2*	5	52,427,696	52,427,819
82	MK	*PHACTR1*	6	13,002,199	13,002,360
83	MK	*PHACTR1*	6	13,019,720	13,019,888
84	MK	*PHACTR1*	6	13,049,651	13,049,938
85	MK	*PHACTR1*	6	13,053,344	13,053,593

86	MK	*PHACTR1*	6	13,055,492	13,056,072
87	MK	*HFE*	6	26,205,599	26,205,740
88	MK	*HFE*	6	26,211,949	26,212,141
89	MK	*HFE*	6	26,231,260	26,231,461
90	MK	*HFE*	6	26,232,120	26,232,359
91	MK	*HFE*	6	26,234,394	26,234,537
92	MK	*BAK1*	6	33,647,044	33,647,233
93	MK	*BAK1*	6	33,655,957	33,656,142
94	MK	*BAK1*	6	33,661,450	33,661,691
95	MK	*MAPK14*	6	36,103,320	36,103,575
96	MK	*MAPK14*	6	36,104,608	36,104,824
97	MK	*MAPK14*	6	36,171,002	36,171,191
98	MK	*MAPK14*	6	36,175,109	36,175,320
99	MK	*MAPK14*	6	36,194,392	36,194,563
100	MK	*MAPK14*	6	36,207,508	36,207,723
101	MK	*BSYL, CCND3*	6	41,996,743	41,997,270
102	MK	*BSYL, CCND3*	6	42,011,971	42,012,241
103	MK	*BSYL, CCND3*	6	42,013,961	42,014,170
104	MK	*BSYL, CCND3*	6	42,015,207	42,015,420
105	MK	*BSYL, CCND3*	6	42,016,385	42,016,594
106	MK	*BSYL, CCND3*	6	42,017,537	42,017,826
107	MK	*BSYL, CCND3*	6	42,093,014	42,093,153
108	MK	*BSYL, CCND3*	6	42,104,341	42,104,544
109	MK	*HBS1L, MYB*	6	135,417,607	135,417,890
110	MK	*HBS1L, MYB*	6	135,456,516	135,456,779
111	MK	*HBS1L, MYB*	6	135,491,460	135,491,619
112	MK	*HBS1L, MYB*	6	135,507,891	135,508,040
113	MK	*HBS1L, MYB*	6	135,543,853	135,544,170
114	MK	*HBS1L, MYB*	6	135,554,328	135,554,777
115	MK	*HBS1L, MYB*	6	135,572,627	135,573,056
116	MK	*HBS1L, MYB*	6	135,575,418	135,575,559
117	MK	*MTHFD1L*	6	151,228,401	151,228,738
118	MK	*MTHFD1L*	6	151,254,856	151,254,996
119	MK	*SLC22A3, LPAL2, LPA*	6	160,797,077	160,797,244
120	MK	*SLC22A3, LPAL2, LPA*	6	160,803,572	160,803,737
121	MK	*SLC22A3, LPAL2, LPA*	6	160,861,000	160,861,377
122	MK	*CD36*	7	80,126,768	80,126,956
123	MK	*FLJ36031, PIK3CG*	7	106,088,749	106,088,894
124	MK	*FLJ36031, PIK3CG*	7	106,145,952	106,146,128
125	MK	*FLJ36031, PIK3CG*	7	106,159,393	106,159,887
126	MK	*AK3, RCL1, JAK2*	9	4,731,175	4,731,296
127	MK	*AK3, RCL1, JAK2*	9	4,740,131	4,740,474
128	MK	*AK3, RCL1, JAK2*	9	4,749,443	4,749,608
129	MK	*AK3, RCL1, JAK2*	9	4,752,645	4,752,818
130	MK	*AK3, RCL1, JAK2*	9	4,782,722	4,782,987

131	MK	*AK3, RCL1, JAK2*	9	4,787,876	4,788,040
132	MK	*AK3, RCL1, JAK2*	9	4,788,379	4,788,594
133	MK	*AK3, RCL1, JAK2*	9	4,789,900	4,790,089
134	MK	*AK3, RCL1, JAK2*	9	4,975,107	4,975,244
135	MK	*AK3, RCL1, JAK2*	9	4,978,008	4,978,177
136	MK	*CACNB2*	10	18,608,247	18,608,466
137	MK	*CACNB2*	10	18,747,666	18,747,838
138	MK	*CXCL12*	10	44,027,150	44,027,346
139	MK	*CXCL12*	10	44,131,962	44,132,213
140	MK	*JMJD1C*	10	64,588,027	64,588,162
141	MK	*JMJD1C*	10	64,596,656	64,596,856
142	MK	*JMJD1C*	10	64,646,953	64,647,146
143	MK	*JMJD1C*	10	64,686,249	64,686,361
144	MK	*JMJD1C*	10	64,694,919	64,695,102
145	MK	*JMJD1C*	10	64,697,313	64,697,676
146	MK	*JMJD1C*	10	64,698,979	64,699,230
147	MK	*JMJD1C*	10	64,818,293	64,818,410
148	MK	*CYP17A1*	10	104,525,551	104,525,930
149	MK	*CYP17A1*	10	104,530,963	104,531,222
150	MK	*CYP17A1*	10	104,546,243	104,546,420
151	MK	*CYP17A1*	10	104,584,763	104,584,934
152	MK	*CYP17A1*	10	104,603,961	104,604,344
153	MK	*CYP17A1*	10	104,651,251	104,651,444
154	MK	*CYP17A1*	10	104,667,304	104,668,027
155	MK	*CYP17A1*	10	104,677,523	104,677,686
156	MK	*BET1L, SIRT3, PSMD13*	11	192,159	192,360
157	MK	*BET1L, SIRT3, PSMD13*	11	197,413	197,708
158	MK	*BET1L, SIRT3, PSMD13*	11	198,637	198,838
159	MK	*BET1L, SIRT3, PSMD13*	11	210,615	210,739
160	MK	*BET1L, SIRT3, PSMD13*	11	226,313	227,021
161	MK	*PLEKHA7*	11	16,798,523	16,798,666
162	MK	*PLEKHA7*	11	16,835,449	16,835,666
163	MK	*PLEKHA7*	11	16,861,395	16,861,589
164	MK	*PLEKHA7*	11	16,914,078	16,914,254
165	MK	*ATP2B1*	12	88,626,699	88,627,268
166	MK	*ATP2B1*	12	88,627,333	88,627,650
167	MK	*SH2B3/LNK, ATXN2*	12	110,319,239	110,319,601
168	MK	*SH2B3/LNK, ATXN2*	12	110,327,583	110,327,960
169	MK	*SH2B3/LNK, ATXN2*	12	110,331,748	110,332,468
170	MK	*SH2B3/LNK, ATXN2*	12	110,351,039	110,351,324
171	MK	*SH2B3/LNK, ATXN2*	12	110,352,457	110,352,655
172	MK	*SH2B3/LNK, ATXN2*	12	110,360,147	110,360,284
173	MK	*SH2B3/LNK, ATXN2*	12	110,360,817	110,360,964
174	MK	*SH2B3/LNK, ATXN2*	12	110,373,957	110,374,307
175	MK	*SH2B3/LNK, ATXN2*	12	110,519,271	110,519,681

176	MK	*SH2B3/LNK, ATXN2*	12	110,521,427	110,521,891
177	MK	*SH2B3/LNK, ATXN2*	12	110,557,836	110,558,035
178	MK	*SH2B3/LNK, ATXN2*	12	110,558,924	110,559,089
179	MK	*PTPN11*	12	111,331,727	111,331,918
180	MK	*PTPN11*	12	111,333,831	111,333,958
181	MK	*PTPN11*	12	111,341,030	111,341,279
182	MK	*PTPN11*	12	111,369,425	111,369,580
183	MK	*TBX3, TBX5*	12	113,844,268	113,844,407
184	MK	*TBX3, TBX5*	12	113,923,933	113,924,094
185	MK	*HNF1A/TCF1, C12orf43*	12	119,894,071	119,894,229
186	MK	*HNF1A/TCF1, C12orf43*	12	119,938,622	119,938,801
187	MK	*HNF1A/TCF1, C12orf43*	12	119,968,834	119,969,061
188	MK	*WDR66*	12	120,761,849	120,762,094
189	MK	*WDR66*	12	120,810,848	120,811,065
190	MK	*WDR66*	12	120,840,714	120,840,925
191	MK	*TPM1*	15	61,121,690	61,121,873
192	MK	*TPM1*	15	61,127,403	61,127,604
193	MK	*TPM1*	15	61,129,502	61,129,701
194	MK	*TPM1*	15	61,130,635	61,130,867
195	MK	*TPM1*	15	61,142,689	61,142,866
196	MK	*TPM1*	15	61,155,658	61,155,927
197	MK	*TPM1*	15	61,158,766	61,158,953
198	MK	*SMAD3*	15	65,160,616	65,160,775
199	MK	*SMAD3*	15	65,170,713	65,170,888
200	MK	*SMAD3*	15	65,177,832	65,178,049
201	MK	*SMAD3*	15	65,189,105	65,189,412
202	MK	*SMAD3*	15	65,237,270	65,237,461
203	MK	*SMAD3*	15	65,287,173	65,287,445
204	MK	*SMAD3*	15	65,334,097	65,334,248
205	MK	*CSK, ULK3*	15	72,856,637	72,856,998
206	MK	*CSK, ULK3*	15	72,859,620	72,859,806
207	MK	*CSK, ULK3*	15	72,866,958	72,867,531
208	MK	*CSK, ULK3*	15	72,922,603	72,922,794
209	MK	*CSK, ULK3*	15	72,947,549	72,947,718
210	MK	*CSK, ULK3*	15	72,949,446	72,949,723
211	MK	*CSK, ULK3*	15	72,952,371	72,952,532
212	MK	*CSK, ULK3*	15	72,969,303	72,969,478
213	MK	*CSK, ULK3*	15	72,986,202	72,986,345
214	MK	*CSK, ULK3*	15	73,017,434	73,017,675
215	MK	*MAP2K4*	17	11,864,740	11,865,023
216	MK	*TAOK1*	17	24,721,381	24,721,534
217	MK	*TAOK1*	17	24,741,338	24,741,554
218	MK	*TAOK1*	17	24,813,063	24,813,280
219	MK	*TAOK1*	17	24,888,222	24,888,373
220	MK	*TAOK1*	17	24,919,622	24,919,734

221	MK	*GSDMA, ORMDL3*	17	35,337,403	35,337,552
222	MK	*GSDMA, ORMDL3*	17	35,362,633	35,363,258
223	MK	*GSDMA, ORMDL3*	17	35,368,822	35,369,018
224	MK	*GSDMA, ORMDL3*	17	35,382,108	35,382,315
225	MK	*CD226*	18	65,657,414	65,657,608
226	MK	*CD226*	18	65,684,550	65,684,718
227	MK	*CD226*	18	65,716,914	65,717,089
228	MK	*MAP2K2*	19	4,040,110	4,040,611
229	MK	*MAP2K2*	19	4,075,128	4,075,297
230	MK	*LDLR*	19	11,060,982	11,061,167
231	MK	*LDLR*	19	11,061,950	11,062,244
232	MK	*AKT2*	19	45,419,804	45,419,969
233	MK	*AKT2*	19	45,483,138	45,483,327
234	MK	*AKT2*	19	45,491,966	45,492,158
235	MK	*AKT2*	19	45,503,197	45,503,389
236	MK	*APOC1, APOC4, APOE*	19	50,041,184	50,041,401
237	MK	*APOC1, APOC4, APOE*	19	50,051,484	50,051,995
238	MK	*APOC1, APOC4, APOE*	19	50,085,805	50,086,330
239	MK	*APOC1, APOC4, APOE*	19	50,150,331	50,150,527
240	MK	*APOC1, APOC4, APOE*	19	50,196,319	50,196,568
241	MK	*APOC1, APOC4, APOE*	19	50,197,042	50,197,263
242	MK	*GP6*	19	60,209,244	60,209,397
243	MK	*GP6*	19	60,241,556	60,241,679
244	MK	*GP6*	19	60,245,094	60,245,382
245	MK	*SIRPA*	20	1,894,347	1,894,752
246	MK	*SLC5A3, MRPS6, KCNE2*	21	34,356,285	34,356,436
247	MK	*SLC5A3, MRPS6, KCNE2*	21	34,367,396	34,367,825
248	MK	*SLC5A3, MRPS6, KCNE2*	21	34,368,044	34,368,422
249	MK	*GNAZ*	22	21,742,217	21,742,604
250	MK	*GNAZ*	22	21,759,106	21,759,267
251	MK	*GNAZ*	22	21,814,206	21,814,411
252	MK	*FBXO7*	22	31,199,429	31,199,582
253	MK	*FBXO7*	22	31,200,572	31,201,245
254	MK	*TMPRSS6*	22	35,734,504	35,734,624
1	EB	*PCSK9*	1	55,274,268	55,274,391
2	EB	*PCSK9*	1	55,277,637	55,277,808
3	EB	*VAV3*	1	107,914,840	107,915,135
4	EB	*CELSR2, PSRC1, SORT1*	1	109,525,340	109,525,629
5	EB	*CELSR2, PSRC1, SORT1*	1	109,537,919	109,538,062
6	EB	*CELSR2, PSRC1, SORT1*	1	109,542,236	109,542,359
7	EB	*CELSR2, PSRC1, SORT1*	1	109,545,395	109,545,538
8	EB	*CELSR2, PSRC1, SORT1*	1	109,557,953	109,558,138
9	EB	*CELSR2, PSRC1, SORT1*	1	109,559,051	109,559,228
10	EB	*CELSR2, PSRC1, SORT1*	1	109,593,547	109,593,856
11	EB	*CELSR2, PSRC1, SORT1*	1	109,598,235	109,598,954

12	EB	*CELSR2, PSRC1, SORT1*	1	109,607,821	109,608,078
13	EB	*CELSR2, PSRC1, SORT1*	1	109,621,701	109,621,870
14	EB	*CELSR2, PSRC1, SORT1*	1	109,627,312	109,627,595
15	EB	*CELSR2, PSRC1, SORT1*	1	109,651,363	109,651,970
16	EB	*CELSR2, PSRC1, SORT1*	1	109,734,038	109,734,237
17	EB	*CELSR2, PSRC1, SORT1*	1	109,737,130	109,737,247
18	EB	*CELSR2, PSRC1, SORT1*	1	109,738,078	109,738,788
19	EB	*PEAR1*	1	155,126,625	155,126,793
20	EB	*PEAR1*	1	155,128,772	155,128,965
21	EB	*PEAR1*	1	155,131,308	155,131,731
22	EB	*PEAR1*	1	155,151,018	155,151,163
23	EB	*PEAR1*	1	155,156,848	155,157,069
24	EB	*PEAR1*	1	155,194,832	155,194,983
25	EB	*FCER1G*	1	159,462,115	159,462,448
26	EB	*FCER1G*	1	159,463,860	159,464,007
27	EB	*DNM3*	1	170,238,636	170,238,785
28	EB	*TMCC2*	1	203,447,052	203,447,525
29	EB	*TMCC2*	1	203,463,544	203,463,687
30	EB	*TMCC2*	1	203,491,827	203,492,110
31	EB	*TMCC2*	1	203,509,317	203,509,504
32	EB	*TMCC2*	1	203,520,592	203,520,795
33	EB	*MIA3*	1	220,695,202	220,695,367
34	EB	*MIA3*	1	220,704,890	220,705,065
35	EB	*MIA3*	1	220,705,358	220,705,709
36	EB	*MIA3*	1	220,829,812	220,829,985
37	EB	*MIA3*	1	220,857,762	220,858,116
38	EB	*MIA3*	1	220,874,539	220,874,712
39	EB	*MIA3*	1	220,884,085	220,884,324
40	EB	*MIA3*	1	220,949,894	220,950,017
41	EB	*MIA3*	1	220,952,051	220,952,194
42	EB	*MIA3*	1	220,952,425	220,953,397
43	EB	*EHD3*	2	31,310,205	31,310,690
44	EB	*STK39*	2	168,701,254	168,701,388
45	EB	*WDR12*	2	203,443,847	203,444,734
46	EB	*WDR12*	2	203,484,326	203,485,130
47	EB	*WDR12*	2	203,485,151	203,485,300
48	EB	*ITPR1*	3	4,831,121	4,831,353
49	EB	*ITPR1*	3	4,842,520	4,842,829
50	EB	*ITPR1*	3	4,847,957	4,848,126
51	EB	*RAF1*	3	12,677,475	12,677,696
52	EB	*RAF1*	3	12,680,631	12,680,868
53	EB	*ULK4*	3	41,716,767	41,716,992
54	EB	*ULK4*	3	41,722,521	41,722,672
55	EB	*ARHGEF3*	3	56,808,646	56,808,811
56	EB	*ARHGEF3*	3	56,810,752	56,811,195

57	EB	*MRAS*	3	139,550,152	139,550,443
58	EB	*P2RY12*	3	152,428,319	152,428,498
59	EB	*P2RY12*	3	152,483,531	152,483,709
60	EB	*P2RY12*	3	152,528,231	152,528,398
61	EB	*ITGA2*	5	52,280,175	52,280,348
62	EB	*ITGA2*	5	52,320,815	52,321,224
63	EB	*ITGA2*	5	52,350,926	52,351,127
64	EB	*ITGA2*	5	52,427,696	52,427,819
65	EB	*PHACTR1*	6	13,002,249	13,002,377
66	EB	*HFE*	6	26,195,745	26,196,200
67	EB	*HFE*	6	26,205,599	26,205,740
68	EB	*HFE*	6	26,211,917	26,212,141
69	EB	*HFE*	6	26,227,238	26,227,731
70	EB	*HFE*	6	26,231,260	26,231,427
71	EB	*HFE*	6	26,232,120	26,232,436
72	EB	*HFE*	6	26,234,394	26,234,537
73	EB	*BAK1*	6	33,647,058	33,647,257
74	EB	*BAK1*	6	33,655,957	33,656,076
75	EB	*MAPK14*	6	36,101,431	36,101,637
76	EB	*MAPK14*	6	36,103,262	36,103,575
77	EB	*MAPK14*	6	36,105,345	36,105,533
78	EB	*MAPK14*	6	36,143,537	36,143,732
79	EB	*MAPK14*	6	36,171,002	36,171,191
80	EB	*MAPK14*	6	36,174,679	36,175,296
81	EB	*MAPK14*	6	36,192,670	36,192,965
82	EB	*MAPK14*	6	36,194,368	36,194,593
83	EB	*MAPK14*	6	36,203,342	36,203,513
84	EB	*BSYL, CCND3*	6	41,996,759	41,997,176
85	EB	*BSYL, CCND3*	6	42,012,097	42,012,241
86	EB	*BSYL, CCND3*	6	42,014,025	42,014,170
87	EB	*BSYL, CCND3*	6	42,015,249	42,015,400
88	EB	*BSYL, CCND3*	6	42,017,009	42,017,658
89	EB	*BSYL, CCND3*	6	42,042,869	42,043,025
90	EB	*BSYL, CCND3*	6	42,043,397	42,043,601
91	EB	*BSYL, CCND3*	6	42,093,014	42,093,185
92	EB	*BSYL, CCND3*	6	42,104,341	42,104,544
93	EB	*HBS1L, MYB*	6	135,417,607	135,417,890
94	EB	*HBS1L, MYB*	6	135,436,982	135,437,159
95	EB	*HBS1L, MYB*	6	135,447,456	135,447,646
96	EB	*HBS1L, MYB*	6	135,457,519	135,457,641
97	EB	*HBS1L, MYB*	6	135,459,977	135,460,413
98	EB	*HBS1L, MYB*	6	135,473,102	135,473,368
99	EB	*HBS1L, MYB*	6	135,507,891	135,508,040
100	EB	*HBS1L, MYB*	6	135,510,588	135,510,958
101	EB	*HBS1L, MYB*	6	135,525,588	135,525,737

102	EB	*HBS1L, MYB*	6	135,543,329	135,543,480
103	EB	*HBS1L, MYB*	6	135,544,020	135,544,170
104	EB	*HBS1L, MYB*	6	135,546,826	135,546,982
105	EB	*HBS1L, MYB*	6	135,554,400	135,555,332
106	EB	*MTHFD1L*	6	151,207,350	151,207,504
107	EB	*MTHFD1L*	6	151,228,401	151,228,738
108	EB	*MTHFD1L*	6	151,254,856	151,254,996
109	EB	*SLC22A3, LPAL2, LPA*	6	160,797,077	160,797,318
110	EB	*SLC22A3, LPAL2, LPA*	6	160,861,000	160,861,409
111	EB	*SLC22A3, LPAL2, LPA*	6	160,919,779	160,919,916
112	EB	*TFR2*	7	100,062,195	100,062,441
113	EB	*TFR2*	7	100,065,016	100,065,141
114	EB	*TFR2*	7	100,077,067	100,077,206
115	EB	*TFR2*	7	100,091,895	100,092,114
116	EB	*FLJ36031, PIK3CG*	7	106,085,915	106,086,298
117	EB	*FLJ36031, PIK3CG*	7	106,086,951	106,087,369
118	EB	*FLJ36031, PIK3CG*	7	106,087,579	106,088,608
119	EB	*FLJ36031, PIK3CG*	7	106,088,749	106,088,894
120	EB	*FLJ36031, PIK3CG*	7	106,106,954	106,107,461
121	EB	*FLJ36031, PIK3CG*	7	106,145,978	106,146,139
122	EB	*FLJ36031, PIK3CG*	7	106,164,291	106,164,480
123	EB	*FLJ36031, PIK3CG*	7	106,165,063	106,165,231
124	EB	*AK3, RCL1, JAK2*	9	4,731,191	4,731,464
125	EB	*AK3, RCL1, JAK2*	9	4,740,131	4,740,281
126	EB	*AK3, RCL1, JAK2*	9	4,752,645	4,752,818
127	EB	*AK3, RCL1, JAK2*	9	4,759,460	4,759,622
128	EB	*AK3, RCL1, JAK2*	9	4,782,722	4,783,059
129	EB	*AK3, RCL1, JAK2*	9	4,788,403	4,788,594
130	EB	*AK3, RCL1, JAK2*	9	4,789,900	4,790,089
131	EB	*AK3, RCL1, JAK2*	9	4,818,810	4,819,123
132	EB	*AK3, RCL1, JAK2*	9	4,823,320	4,823,517
133	EB	*AK3, RCL1, JAK2*	9	4,842,517	4,842,710
134	EB	*AK3, RCL1, JAK2*	9	4,845,071	4,845,268
135	EB	*AK3, RCL1, JAK2*	9	4,852,849	4,853,186
136	EB	*AK3, RCL1, JAK2*	9	4,855,814	4,855,959
137	EB	*AK3, RCL1, JAK2*	9	4,914,359	4,914,516
138	EB	*AK3, RCL1, JAK2*	9	4,917,093	4,917,637
139	EB	*AK3, RCL1, JAK2*	9	4,975,147	4,975,388
140	EB	*AK3, RCL1, JAK2*	9	4,978,008	4,978,131
141	EB	*CXCL12*	10	44,126,559	44,126,820
142	EB	*CXCL12*	10	44,131,944	44,132,213
143	EB	*JMJD1C*	10	64,588,027	64,588,162
144	EB	*JMJD1C*	10	64,596,732	64,596,856
145	EB	*JMJD1C*	10	64,646,971	64,647,116
146	EB	*JMJD1C*	10	64,666,261	64,666,430

147	EB	*JMJD1C*	10	64,694,262	64,694,665
148	EB	*JMJD1C*	10	64,694,963	64,695,179
149	EB	*JMJD1C*	10	64,695,295	64,696,104
150	EB	*JMJD1C*	10	64,696,220	64,697,726
151	EB	*JMJD1C*	10	64,697,743	64,698,439
152	EB	*JMJD1C*	10	64,698,979	64,699,206
153	EB	*JMJD1C*	10	64,753,836	64,754,060
154	EB	*JMJD1C*	10	64,894,834	64,895,217
155	EB	*JMJD1C*	10	64,895,652	64,895,891
156	EB	*CYP17A1*	10	104,564,250	104,564,529
157	EB	*CYP17A1*	10	104,604,251	104,604,420
158	EB	*CYP17A1*	10	104,651,251	104,651,444
159	EB	*CYP17A1*	10	104,667,738	104,668,027
160	EB	*BET1L, SIRT3, PSMD13*	11	197,021	197,756
161	EB	*BET1L, SIRT3, PSMD13*	11	210,615	210,739
162	EB	*BET1L, SIRT3, PSMD13*	11	226,313	226,941
163	EB	*PLEKHA7*	11	16,798,523	16,798,666
164	EB	*PLEKHA7*	11	16,835,449	16,835,666
165	EB	*ATP2B1*	12	88,460,946	88,461,129
166	EB	*ATP2B1*	12	88,492,709	88,493,151
167	EB	*ATP2B1*	12	88,581,495	88,581,644
168	EB	*ATP2B1*	12	88,582,070	88,582,255
169	EB	*ATP2B1*	12	88,596,236	88,596,392
170	EB	*ATP2B1*	12	88,626,553	88,627,268
171	EB	*ATP2B1*	12	88,627,333	88,627,650
172	EB	*SH2B3/LNK, ATXN2*	12	110,325,149	110,325,300
173	EB	*SH2B3/LNK, ATXN2*	12	110,327,583	110,328,060
174	EB	*SH2B3/LNK, ATXN2*	12	110,332,012	110,332,542
175	EB	*SH2B3/LNK, ATXN2*	12	110,373,983	110,374,130
176	EB	*SH2B3/LNK, ATXN2*	12	110,500,460	110,500,606
177	EB	*SH2B3/LNK, ATXN2*	12	110,519,271	110,519,415
178	EB	*SH2B3/LNK, ATXN2*	12	110,520,341	110,521,038
179	EB	*SH2B3/LNK, ATXN2*	12	110,521,760	110,521,891
180	EB	*SH2B3/LNK, ATXN2*	12	110,557,836	110,557,985
181	EB	*SH2B3/LNK, ATXN2*	12	110,558,848	110,559,043
182	EB	*PTPN11*	12	111,331,727	111,331,862
183	EB	*PTPN11*	12	111,333,831	111,333,979
184	EB	*PTPN11*	12	111,340,934	111,341,279
185	EB	*HNF1A/TCF1, C12orf43*	12	119,894,015	119,894,229
186	EB	*HNF1A/TCF1, C12orf43*	12	119,938,622	119,938,801
187	EB	*WDR66*	12	120,761,821	120,762,094
188	EB	*WDR66*	12	120,810,814	120,811,511
189	EB	*WDR66*	12	120,815,510	120,815,633
190	EB	*WDR66*	12	120,856,177	120,856,394
191	EB	*WDR66*	12	120,944,486	120,945,014

192	EB	*TPM1*		15	61,127,313	61,127,676
193	EB	*TPM1*		15	61,130,687	61,130,867
194	EB	*TPM1*		15	61,142,505	61,142,866
195	EB	*SMAD3*		15	65,155,728	65,155,873
196	EB	*SMAD3*		15	65,170,689	65,170,888
197	EB	*SMAD3*		15	65,205,049	65,205,218
198	EB	*SMAD3*		15	65,287,103	65,287,445
199	EB	*SMAD3*		15	65,334,079	65,334,248
200	EB	*CSK, ULK3*		15	72,861,346	72,861,599
201	EB	*CSK, ULK3*		15	72,922,029	72,922,288
202	EB	*CSK, ULK3*		15	72,922,603	72,922,794
203	EB	*CSK, ULK3*		15	72,952,395	72,952,532
204	EB	*CSK, ULK3*		15	72,969,359	72,969,478
205	EB	*CSK, ULK3*		15	72,986,226	72,986,345
206	EB	*CSK, ULK3*		15	73,017,434	73,017,675
207	EB	*CSK, ULK3*		15	73,029,865	73,030,026
208	EB	*MAP2K4*		17	11,864,740	11,864,995
209	EB	*TAOK1*		17	24,721,397	24,721,534
210	EB	*TAOK1*		17	24,741,312	24,741,554
211	EB	*TAOK1*		17	24,888,250	24,888,373
212	EB	*TAOK1*		17	24,908,398	24,908,777
213	EB	*TAOK1*		17	24,919,092	24,919,239
214	EB	*TAOK1*		17	24,919,646	24,919,832
215	EB	*GSDMA, ORMDL3*		17	35,337,403	35,337,552
216	EB	*GSDMA, ORMDL3*		17	35,339,061	35,339,338
217	EB	*GSDMA, ORMDL3*		17	35,362,511	35,363,278
218	EB	*GSDMA, ORMDL3*		17	35,368,822	35,368,959
219	EB	*CD226*		18	65,657,222	65,657,632
220	EB	*MAP2K2*		19	4,075,128	4,075,297
221	EB	*MAP2K2*		19	4,082,054	4,082,195
222	EB	*LDLR*		19	11,061,022	11,061,167
223	EB	*LDLR*		19	11,062,115	11,062,260
224	EB	*LDLR*		19	11,066,803	11,066,988
225	EB	*AKT2*		19	45,406,952	45,407,150
226	EB	*AKT2*		19	45,483,138	45,483,327
227	EB	*AKT2*		19	45,503,197	45,503,389
228	EB	*APOC1, APOC4, APOE*		19	50,041,082	50,041,319
229	EB	*APOC1, APOC4, APOE*		19	50,085,593	50,086,330
230	EB	*APOC1, APOC4, APOE*		19	50,122,302	50,122,487
231	EB	*APOC1, APOC4, APOE*		19	50,150,303	50,150,503
232	EB	*APOC1, APOC4, APOE*		19	50,196,398	50,196,568
233	EB	*APOC1, APOC4, APOE*		19	50,197,042	50,197,351
234	EB	*GP6*		19	60,209,244	60,209,397
235	EB	*GP6*		19	60,230,073	60,230,138
236	EB	*GP6*		19	60,230,479	60,230,694

237	EB	*GP6*	19	60,241,578	60,241,731
238	EB	*GP6*	19	60,245,122	60,245,382
239	EB	*SIRPA*	20	1,823,147	1,824,032
240	EB	*SIRPA*	20	1,894,417	1,894,616
241	EB	*SLC5A3, MRPS6, KCNE2*	21	34,356,285	34,356,436
242	EB	*SLC5A3, MRPS6, KCNE2*	21	34,367,396	34,367,825
243	EB	*SLC5A3, MRPS6, KCNE2*	21	34,368,044	34,368,452
244	EB	*SLC5A3, MRPS6, KCNE2*	21	34,404,980	34,405,117
245	EB	*SLC5A3, MRPS6, KCNE2*	21	34,484,864	34,485,014
246	EB	*GNAZ*	22	21,742,291	21,742,604
247	EB	*GNAZ*	22	21,814,182	21,814,349
248	EB	*GNAZ*	22	21,817,463	21,817,638
249	EB	*FBXO7*	22	31,195,930	31,196,041
250	EB	*FBXO7*	22	31,200,572	31,200,839
251	EB	*FBXO7*	22	31,208,867	31,209,065

**(B) Lineage-specific reference genes**						
**#**	**Cell type**	**Gene**	**Lineage**	**Genomic position**		
				**Chromosome**	**Start**	**End**
1	MK	*SELP*	megakaryocytic	1	167,837,047	167,837,214
2	MK	*SELP*	megakaryocytic	1	167,840,713	167,840,910
3	MK	*SELP*	megakaryocytic	1	167,860,923	167,861,131
4	MK	*SELP*	megakaryocytic	1	167,864,661	167,864,809
5	MK	*SELP*	megakaryocytic	1	167,865,949	167,866,124
6	MK	*DDEF2*	megakaryocytic	2	9,263,856	9,264,613
7	MK	*DDEF2*	megakaryocytic	2	9,280,894	9,281,103
8	MK	*DDEF2*	megakaryocytic	2	9,288,553	9,288,734
9	MK	*DDEF2*	megakaryocytic	2	9,292,403	9,292,583
10	MK	*DDEF2*	megakaryocytic	2	9,321,027	9,321,370
11	MK	*DDEF2*	megakaryocytic	2	9,324,449	9,324,618
12	MK	*DDEF2*	megakaryocytic	2	9,352,468	9,352,664
13	MK	*DDEF2*	megakaryocytic	2	9,388,234	9,388,453
14	MK	*DDEF2*	megakaryocytic	2	9,389,371	9,389,510
15	MK	*DDEF2*	megakaryocytic	2	9,391,309	9,391,498
16	MK	*MEIS1*	megakaryocytic	2	66,514,062	66,514,325
17	MK	*MEIS1*	megakaryocytic	2	66,514,948	66,515,087
18	MK	*MEIS1*	megakaryocytic	2	66,515,690	66,515,914
19	MK	*MEIS1*	megakaryocytic	2	66,559,525	66,559,685
20	MK	*MEIS1*	megakaryocytic	2	66,561,771	66,562,180
21	MK	*MEIS1*	megakaryocytic	2	66,570,205	66,570,361
22	MK	*MEIS1*	megakaryocytic	2	66,571,513	66,571,665
23	MK	*MEIS1*	megakaryocytic	2	66,607,872	66,607,998
24	MK	*MEIS1*	megakaryocytic	2	66,608,356	66,608,553
25	MK	*PID1*	monocytic	2	229,753,747	229,754,002

26	MK	*MYLK*	megakaryocytic	3	124,844,018	124,844,185
27	MK	*MYLK*	megakaryocytic	3	124,869,318	124,869,576
28	MK	*MYLK*	megakaryocytic	3	124,895,526	124,895,719
29	MK	*MYLK*	megakaryocytic	3	124,897,280	124,897,593
30	MK	*MYLK*	megakaryocytic	3	124,925,235	124,925,374
31	MK	*MYLK*	megakaryocytic	3	125,015,596	125,015,865
32	MK	*MYLK*	megakaryocytic	3	125,043,039	125,043,245
33	MK	*MYLK*	megakaryocytic	3	125,085,915	125,086,098
34	MK	*FAM83A*	erythroblastoid	8	124,263,976	124,264,163
35	MK	*FAM83A*	erythroblastoid	8	124,284,318	124,284,538
36	MK	*NFIB*	megakaryocytic	9	14,145,441	14,145,629
37	MK	*NFIB*	megakaryocytic	9	14,189,398	14,189,555
38	MK	*NFIB*	megakaryocytic	9	14,200,125	14,200,850
39	MK	*NFIB*	megakaryocytic	9	14,202,185	14,203,126
40	MK	*NFIB*	megakaryocytic	9	14,204,486	14,204,659
41	MK	*NFIB*	megakaryocytic	9	14,299,554	14,299,728
42	MK	*KLF4*	monocytic	9	109,286,348	109,286,517
43	MK	*FER1L3*	monocytic	10	95,102,486	95,102,667
44	MK	*FER1L3*	monocytic	10	95,107,552	95,107,709
45	MK	*FER1L3*	monocytic	10	95,126,230	95,126,399
46	MK	*FER1L3*	monocytic	10	95,162,999	95,163,416
47	MK	*FER1L3*	monocytic	10	95,185,908	95,186,083
48	MK	*FER1L3*	monocytic	10	95,192,142	95,192,393
49	MK	*FER1L3*	monocytic	10	95,198,267	95,198,447
50	MK	*FER1L3*	monocytic	10	95,232,410	95,232,551
51	MK	*ADCY6*	megakaryocytic	12	47,445,610	47,446,237
52	MK	*ADCY6*	megakaryocytic	12	47,460,026	47,460,291
53	MK	*CMTM5*	megakaryocytic	14	22,916,720	22,916,905
54	MK	*CALB2*	erythroblastoid	16	69,949,952	69,950,141
55	MK	*CALB2*	erythroblastoid	16	69,958,830	69,959,111
56	MK	*GDF15*	erythroblastoid	19	18,360,289	18,360,482
57	MK	*RIN2*	monocytic	20	19,863,502	19,863,781
58	MK	*RIN2*	monocytic	20	19,871,823	19,871,962
59	MK	*RIN2*	monocytic	20	19,924,018	19,924,243
1	EB	*DDEF2*	megakaryocytic	2	9,263,856	9,264,367
2	EB	*DDEF2*	megakaryocytic	2	9,292,425	9,292,593
3	EB	*DDEF2*	megakaryocytic	2	9,389,371	9,389,510
4	EB	*DDEF2*	megakaryocytic	2	9,392,990	9,393,351
5	EB	*DDEF2*	megakaryocytic	2	9,394,746	9,394,892
6	EB	*MEIS1*	megakaryocytic	2	66,514,142	66,514,283
7	EB	*MEIS1*	megakaryocytic	2	66,515,690	66,515,914
8	EB	*MEIS1*	megakaryocytic	2	66,561,947	66,562,116
9	EB	*MEIS1*	megakaryocytic	2	66,571,277	66,571,637
10	EB	*MEIS1*	megakaryocytic	2	66,576,847	66,577,049
11	EB	*LOC51252*	erythroblastoid	2	96,924,685	96,924,852

12	EB	*PID1*	monocytic	2	229,753,747	229,754,002
13	EB	*MYLK*	megakaryocytic	3	124,838,096	124,838,321
14	EB	*MYLK*	megakaryocytic	3	124,844,018	124,844,185
15	EB	*MYLK*	megakaryocytic	3	124,925,235	124,925,374
16	EB	*MYLK*	megakaryocytic	3	125,085,779	125,086,098
17	EB	*CA1*	erythroblastoid	8	86,477,702	86,477,887
18	EB	*FAM83A*	erythroblastoid	8	124,264,050	124,264,163
19	EB	*NFIB*	megakaryocytic	9	14,098,110	14,098,253
20	EB	*NFIB*	megakaryocytic	9	14,303,979	14,304,498
21	EB	*KLF4*	monocytic	9	109,286,348	109,286,487
22	EB	*KLF4*	monocytic	9	109,291,850	109,292,093
23	EB	*FER1L3*	monocytic	10	95,102,464	95,102,667
24	EB	*FER1L3*	monocytic	10	95,232,428	95,232,551
25	EB	*EPB42*	erythroblastoid	15	41,300,451	41,300,890
26	EB	*HBZ*	erythroblastoid	16	142,536	142,677
27	EB	*HBZ*	erythroblastoid	16	144,102	144,258
28	EB	*ERAF*	erythroblastoid	16	31,446,616	31,446,727
29	EB	*CALB2*	erythroblastoid	16	69,958,830	69,958,999
30	EB	*GDF15*	erythroblastoid	19	18,357,853	18,358,148
31	EB	*GDF15*	erythroblastoid	19	18,360,091	18,360,482
32	EB	*RIN2*	monocytic	20	19,876,616	19,876,811
33	EB	*RIN2*	monocytic	20	19,924,062	19,924,380
