# Supplementary material for: Maps of Open Chromatin Guide the Functional Follow-Up of Genome-Wide Association Signals: Application to Hematological Traits
Source: PLoS Genet. 2011 Jun 30;7(6):e1002139. doi: 10.1371/journal.pgen.1002139 (PMC3128100; doi:10.1371/journal.pgen.1002139)
Supplement: Table S2 — Comparison of the FAIRE peak density between the ENCODE and the here presented data sets. (PDF) [file pgen.1002139.s009.pdf]

Table S2. Comparison of the FAIRE peak density between the ENCODE (#1) and the here presented data sets (#2a,b).

#	Human cell line	Formaldehyde cross-linking	Total sequence	Total number of peaks	Number of peaks per Mb
1	Foreskin fibroblasts [CCD-1070Sk]	7 min	29,998 kb	1,008	33.6
2a	MK cells [CHRF-288-11]	8 min	9,651 kb	397	41.1
		12 min		364	37.7
2b	EB cells [K562]	8 min	9,651 kb	376	39.0
		12 min		333	34.5
