# Supplementary material for: Maps of Open Chromatin Guide the Functional Follow-Up of Genome-Wide Association Signals: Application to Hematological Traits
Source: PLoS Genet. 2011 Jun 30;7(6):e1002139. doi: 10.1371/journal.pgen.1002139 (PMC3128100; doi:10.1371/journal.pgen.1002139)
Supplement: Table S3 — Characterization of open chromatin regions in relation to gene annotations. (PDF) [file pgen.1002139.s010.pdf]

Table S3. Characterization of open chromatin regions in relation to gene annotations.

Genomic location	MK cells only	EB cells only	Both cell types
Intergenic	20.2%	39.3%	26.5%
Intronic	72.5%	45.8%	41.4%
Overlap 3'-UTR	0.9%	1.9%	1.9%
Overlap 5'-UTR	4.6%	5.6%	28.4%
Exonic	1.8%	7.5%	1.9%
