# Supplementary material for: Maps of Open Chromatin Guide the Functional Follow-Up of Genome-Wide Association Signals: Application to Hematological Traits
Source: PLoS Genet. 2011 Jun 30;7(6):e1002139. doi: 10.1371/journal.pgen.1002139 (PMC3128100; doi:10.1371/journal.pgen.1002139)
Supplement: Table S4 — Investigation of the functional role of platelet volume-associated variants at chromosome 7q22.3. (PDF) [file pgen.1002139.s011.pdf]

Table S4. Investigation of the functional role of platelet volume-associated variants at chromosome 7q22.3.

Proxy to rs342293							SNP overlaps with …		
ID	Chromosome	Position	r^2	Distance	Annotation	MPV *P*-value	NDR in MK cells	Transcription factor (TF) binding site (MatInspector)	Binding site of TF expressed in MK cells
rs342207	7	106,108,641	0.81	50,814	Intergenic	1.46E-10	-	-	-
rs342209	7	106,109,208	0.84	50,247	Intergenic	NA	-	-	-
rs342210	7	106,109,256	0.87	50,199	Intergenic	3.57E-11	-	-	-
rs342212	7	106,111,150	0.84	48,305	Intergenic	5.47E-11	-	-	-
rs342213	7	106,111,848	0.84	47,607	Intergenic	6.21E-11	-	-	-
rs342214	7	106,111,979	0.81	47,476	Intergenic	NA	-	-	-
rs342236	7	106,122,874	0.84	36,581	Intergenic	1.24E-10	-	-	-
rs342239	7	106,124,138	0.90	35,317	Intergenic	6.97E-11	-	-	-
rs342240	7	106,124,486	0.90	34,969	Intergenic	6.97E-11	-	HMX2	-
rs342241	7	106,124,587	0.90	34,868	Intergenic	NA	-	-	-
rs342242	7	106,126,225	0.90	33,230	Intergenic	6.43E-11	-	-	-
rs342244	7	106,128,061	0.81	31,394	Intergenic	1.43E-10	-	-	-
rs342247	7	106,130,427	0.84	29,028	Intergenic	4.23E-11	-	HHEX, HOXC4, LBX2, MSX	HHEX, MSX
rs342248	7	106,130,541	0.84	28,914	Intergenic	NA	-	-	-
rs342251	7	106,132,045	0.90	27,410	Intergenic	NA	-	-	-
rs342252	7	106,133,666	0.90	25,789	Intergenic	NA	-	-	-
rs342254	7	106,135,492	0.90	23,963	Intergenic	3.84E-11	-	-	-
rs342257	7	106,137,089	0.84	22,366	Intergenic	6.09E-11	-	-	-

rs342271	7	106,143,111	0.90	16,344	Intergenic	NA	-	-	-
rs342275	7	106,146,452	0.90	13,003	Intergenic	1.33E-11	-	-	-
rs342281	7	106,149,079	0.84	10,376	Intergenic	5.36E-12	-	-	-
rs342284	7	106,149,446	0.87	10,009	Intergenic	4.62E-12	-	-	-
rs342286	7	106,151,835	0.94	7,620	Intergenic	4.90E-12	-	-	-
rs342290	7	106,154,840	1.00	4,615	Intergenic	NA	-	-	-
rs342292	7	106,157,880	1.00	1,575	Intergenic	8.64E-13	-	MEIS1, MEIS1A/HOXA9	MEIS1, MEIS1A/HOXA9
rs342293	7	106,159,455	1.00	0	Intergenic	6.75E-13	**+**	GATA1, EVI1	GATA1, EVI1
rs342294	7	106,159,858	1.00	403	Intergenic	NA	**+**	-	-
rs342295	7	106,159,996	1.00	541	Intergenic	4.14E-13	-	-	-
rs342296	7	106,160,139	1.00	684	Intergenic	7.68E-13	-	-	-
rs342298	7	106,160,882	0.97	1,427	Intergenic	7.55E-13	-	-	-
rs342299	7	106,160,954	0.97	1,499	Intergenic	7.55E-13	-	-	-
rs386805	7	106,125,700	0.97	33,755	Intergenic	NA	-	-	-
rs67036916	7	106,154,872	0.97	4,583	Intergenic	NA	-	-	-
rs77655772	7	106,125,699	0.97	33,756	Intergenic	NA	-	-	-

Proxy-SNPs to rs342293 ($r^2 \geq 0.8$) were retrieved from the 1000 Genomes Project (Pilot 1, CEU). Genomic coordinates are based on the human reference genome, build hg18 (NCBI build 36). *P*-values for association with mean platelet volume (MPV) were obtained from Soranzo et al. *In silico* transcription binding site predictions were performed as described (Materials and Methods). Based on the HaemAtlas data, we defined genes as expressed when they exhibit a normalized expression value of at least 8.5.
