# Supplementary material for: Maps of Open Chromatin Guide the Functional Follow-Up of Genome-Wide Association Signals: Application to Hematological Traits
Source: PLoS Genet. 2011 Jun 30;7(6):e1002139. doi: 10.1371/journal.pgen.1002139 (PMC3128100; doi:10.1371/journal.pgen.1002139)
Supplement: Table S5 — Resequencing of the MK-specific open chromatin region at chromosome 7q22.3. (PDF) [file pgen.1002139.s012.pdf]

Table S5. Resequencing of the MK-specific open chromatin region at chromosome 7q22.3.

	**rs342293**	***private***	**rs342294**
Calls	600/643 (93.3%)	633/643 (98.4%)	330/643 (51.3%)
Position	chr7:106,159,455	chr7:106,159,601	chr7:106,159,858
Genotype	C/G	A/C	T/C
MAF (observed)	0.4167	0.0008	0.3909
MAF (HapMap)	0.482	N/A	0.482
MAF (1K Genomes)	0.458	N/A	0.458
Sequencing traces	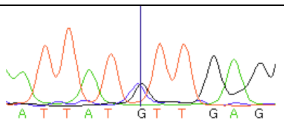	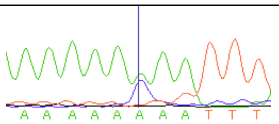	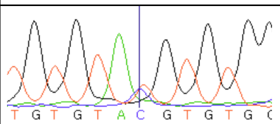

The low call rate for rs342294 was due to the location of the SNP, at the very end of the STS. Minor allele frequencies (MAF) from Phase II+III HapMap (CEU) and the 1000 Genomes Project (Pilot 1, CEU) were extracted from dbSNP v131. Genomic coordinates are based on the human reference genome, build hg18.
