# Supplementary material for: Maps of Open Chromatin Guide the Functional Follow-Up of Genome-Wide Association Signals: Application to Hematological Traits
Source: PLoS Genet. 2011 Jun 30;7(6):e1002139. doi: 10.1371/journal.pgen.1002139 (PMC3128100; doi:10.1371/journal.pgen.1002139)
Supplement: Table S6 — Expression QTL associations at the PIK3CG gene locus in platelets, macrophages, monocytes, B cells (LCLs), fat, and skin. (PDF) [file pgen.1002139.s013.pdf]

Table S6. Expression QTL associations at the *PIK3CG* gene locus in platelets, macrophages, monocytes, B cells (LCLs), fat and skin.

Cell type / tissue	GWA index SNP	Proxy to rs342293			eQTL nominal *P*-value	SNP with the strongest association with *PIK3CG* expression in 1-Mb window		
		ID	r^2	Distance		ID	r^2 with rs342293	eQTL nominal *P*-value
Platelets	rs342293				0.0542	rs342293	1.000	
Macrophages		rs342275	0.935	13,003 bp	0.0018	rs342275	0.935	
Monocytes		rs342275	0.935	13,003 bp	0.4348	rs10953522	0.002	0.0005
LCLs		rs342296	1.000	684 bp	0.5983	rs7788626	0.032	0.0018
		rs342275	0.935	13,003 bp	0.9999			
Fat		rs342296	1.000	684 bp	0.5308	rs849375	0.059	0.0022
		rs342275	0.935	13,003 bp	0.6917			
Skin		rs342296	1.000	684 bp	0.2091	rs13246564	0.045	0.0006
		rs342275	0.935	13,003 bp	0.2537			

The strength of the relationship between alleles and gene expression intensities was estimated with the Spearman's rank correlation coefficient using the software Genevar (Materials and Methods). In macrophages and monocytes (as part of the data generated by the Cardiogenics Consortium), the rs342293 proxy-SNP rs342275 was used for the eQTL analysis. For LCLs, fat and skin (as part of the data generated by the MuTHER Consortium), the proxy-SNPs rs342296 and rs342275 were used for analysis. Values for r^2 were obtained from Phase II HapMap, CEU.
