# Supplementary material for: Maps of Open Chromatin Guide the Functional Follow-Up of Genome-Wide Association Signals: Application to Hematological Traits
Source: PLoS Genet. 2011 Jun 30;7(6):e1002139. doi: 10.1371/journal.pgen.1002139 (PMC3128100; doi:10.1371/journal.pgen.1002139)
Supplement: Table S7 — Differentially expressed genes between Pik3cg−/− and wild-type mice. (PDF) [file pgen.1002139.s014.pdf]

Table S7. Differentially expressed genes between *Pik3cg*$^{-/-}$ and wild type mice.

#	Probe-ID	Gene	*Pik3cg*$^{-/-}$ (n=3)		Wild type (n=3)		log_2-FC	FC	*P*-value
			Mean	sd	Mean	sd			
1	2635272	*Igh-VJ558*	10.9383	0.4107	8.6536	0.2362	2.285	4.873	1.123E-03
2	2846865	*Actb*	10.7849	0.3334	8.5469	0.1962	2.238	4.717	5.576E-04
3	2588055	*Actb*	10.6790	0.2479	8.5161	0.1759	2.163	4.478	2.490E-04
4	1226402	*LOC331507*	11.0561	0.4100	8.9195	0.2245	2.137	4.397	1.377E-03
5	2648661	*Cap1*	9.8324	0.1852	7.8609	0.0746	1.972	3.922	6.859E-05
6	2660754	*Spnb1*	11.5861	0.2838	10.0427	0.1154	1.543	2.915	9.499E-04
7	2434853	*mtDNA_ND2*	11.9292	0.3324	10.5779	0.4537	1.351	2.551	1.413E-02
8	2772264	*Igh-VJ558*	9.4057	0.1730	8.0732	0.1451	1.333	2.518	5.161E-04
9	1230858	*9430065F12Rik*	10.5905	0.3804	9.2592	0.0696	1.331	2.516	3.972E-03
10	2624477	*E2f2*	10.4032	0.1284	9.2063	0.0898	1.197	2.293	1.884E-04
11	1216143	*Rnf11*	13.7329	0.2317	12.5869	0.1436	1.146	2.213	1.888E-03
12	1249344	*2510006D16Rik*	9.4496	0.9088	8.3631	0.0232	1.086	2.124	1.072E-01
13	2705407	*Pabpc1*	9.2546	0.6745	8.1683	0.1240	1.086	2.123	5.171E-02
14	1214408	*LOC666403*	9.7787	1.9423	8.7006	0.0809	1.078	2.111	3.911E-01
15	1257767	*E430014K09Rik*	8.8017	0.0575	7.7333	0.0294	1.068	2.097	8.860E-06
16	1233929	*Psmf1*	13.1145	0.1714	12.0549	0.0801	1.060	2.084	6.318E-04
17	2720813	*Epb4.1*	11.5693	0.4973	10.5321	0.2065	1.037	2.052	2.894E-02
18	3114413	*G3bp2*	11.4849	0.3002	10.4546	0.1420	1.030	2.042	5.794E-03
19	1241268	*Snx3*	10.8379	0.2128	9.8196	0.1785	1.018	2.026	3.150E-03
20	2698430	*Bcl2l1*	9.8201	0.7633	8.8199	0.2164	1.000	2.000	9.436E-02
21	2683128	*Cmas*	13.7820	0.4440	12.7996	0.3039	0.982	1.976	3.409E-02
22	2459979	*Fancl*	9.0993	0.2659	8.1226	0.0528	0.977	1.968	3.362E-03
23	2911760	*Ubac1*	11.6110	0.1664	10.6409	0.2460	0.970	1.959	4.812E-03
24	2666747	*G3bp2*	9.2567	0.4938	8.3037	0.0733	0.953	1.936	2.976E-02
25	2632206	*Gnas*	10.6836	0.4952	9.7392	0.1018	0.944	1.924	3.182E-02
26	2894450	*Snx15*	11.7147	0.2518	10.7811	0.2630	0.934	1.910	1.132E-02
27	1260103	*LOC666904*	14.3019	0.3975	13.3887	0.2117	0.913	1.883	2.463E-02
28	2969919	*Rnf11*	14.5588	0.2325	13.6472	0.1255	0.912	1.881	3.939E-03
29	2453695	*Urod*	12.6295	0.3807	11.7423	0.0647	0.887	1.850	1.641E-02
30	2622671	*Acsl1*	9.3216	0.4188	8.4621	0.1160	0.860	1.815	2.663E-02
31	2588411	*Grina*	15.1590	0.0817	14.3097	0.1321	0.849	1.802	6.938E-04
32	1255562	*Fancl*	8.9004	0.1910	8.0561	0.0449	0.844	1.795	1.730E-03
33	2962747	*Fzr1*	11.9614	0.2804	11.1277	0.2226	0.834	1.782	1.569E-02
34	2956895	*Cul4a*	10.6666	0.2442	9.8359	0.1071	0.831	1.779	5.711E-03
35	1259769	*Pabpc1*	9.5486	0.4774	8.7251	0.1611	0.824	1.770	4.729E-02
36	3116560	*St3gal5*	10.8780	0.2258	10.0574	0.1030	0.821	1.766	4.601E-03
37	1213070	*1700037H04Rik*	11.8912	0.2351	11.0714	0.1019	0.820	1.765	5.185E-03
38	2939295	*Fis1*	12.2089	0.5195	11.4108	0.1927	0.798	1.739	6.713E-02
39	2713686	*Pnpo*	9.0831	0.7739	8.2924	0.1096	0.791	1.730	1.546E-01
40	2736762	*Ifrd2*	9.5116	0.2988	8.7241	0.1702	0.788	1.726	1.657E-02
41	2746830	*BC022224*	13.8552	0.1850	13.0779	0.2047	0.777	1.714	8.161E-03

42	2944601	*4933439C20Rik*	9.9683	0.2884	9.1956	0.0777	0.773	1.708	1.099E-02
43	2655260	*Ptp4a3*	11.0441	0.2817	10.2739	0.1685	0.770	1.706	1.529E-02
44	1217767	*Glrx5*	14.9998	0.1628	14.2389	0.1197	0.761	1.695	2.855E-03
45	2855792	*Pnpo*	11.2950	0.2852	10.5477	0.1420	0.747	1.679	1.532E-02
46	3026397	*Chka*	11.0370	0.0709	10.2926	0.1943	0.744	1.675	3.378E-03
47	1226372	*Rffl*	9.3176	0.1234	8.5745	0.1069	0.743	1.674	1.400E-03
48	2939294	*Fis1*	11.8499	0.3965	11.1082	0.2618	0.742	1.672	5.388E-02
49	2770819	*Rnf10*	10.4975	0.4380	9.7562	0.0606	0.741	1.672	4.393E-02
50	2431046	*Ubac1*	10.3293	0.2206	9.5890	0.2027	0.740	1.671	1.284E-02
51	1236577	*2310045N01Rik*	9.4111	0.0609	8.6769	0.0754	0.734	1.664	1.949E-04
52	2691567	*Hyi*	8.7761	0.1367	8.0449	0.1035	0.731	1.660	1.790E-03
53	2958159	*Eno1*	9.2303	0.2476	8.5026	0.0188	0.728	1.656	7.096E-03
54	2694782	*2410076I21Rik*	14.6397	0.1542	13.9149	0.0294	0.725	1.653	1.325E-03
55	2677712	*Arf5*	9.5897	0.5985	8.8664	0.1245	0.723	1.651	1.098E-01
56	1234387	*Acp1*	8.7540	0.4122	8.0571	0.1525	0.697	1.621	5.154E-02
57	2940642	*St6galnac2*	8.4426	0.1862	7.7463	0.1026	0.696	1.620	4.763E-03
58	2656854	*Myo6*	9.1095	0.1882	8.4192	0.0592	0.690	1.614	3.745E-03
59	2483948	*Specc1*	13.0098	0.1772	12.3245	0.1838	0.685	1.608	9.663E-03
60	1241976	*Ubac1*	9.6326	0.2404	8.9499	0.1999	0.683	1.605	1.941E-02
61	2860699	*Epb4.9*	9.5381	0.1654	8.8585	0.1222	0.680	1.602	4.614E-03
62	2551648	*Mscp-pending*	8.9890	0.1299	8.3099	0.0170	0.679	1.601	8.513E-04
63	2652187	*Cux1*	10.3852	0.2232	9.7106	0.2137	0.675	1.596	1.942E-02
64	2767918	*Ifi30*	9.2217	0.0853	8.5497	0.0969	0.672	1.593	8.379E-04
65	1228366	*Cdkn2c*	9.7009	0.2510	9.0289	0.1665	0.672	1.593	1.808E-02
66	2606746	*Car2*	9.7239	0.5358	9.0531	0.2742	0.671	1.592	1.258E-01
67	2653765	*Nusap1*	12.6153	0.3736	11.9481	0.2863	0.667	1.588	7.003E-02
68	1249578	*Sorl1*	8.9404	0.1175	8.2735	0.0482	0.667	1.588	8.094E-04
69	2605453	*Ranbp10*	12.0146	0.1143	11.3487	0.0177	0.666	1.586	5.680E-04
70	2735615	*Isg20*	11.5303	0.1583	10.8841	0.2082	0.646	1.565	1.285E-02
71	1217068	*Tusc1*	9.9727	0.1396	9.3287	0.1806	0.644	1.563	8.124E-03
72	2445324	*LOC100047651*	10.1668	0.0549	9.5257	0.0439	0.641	1.559	9.365E-05
73	1250068	*Atpif1*	12.3942	0.3794	11.7544	0.3283	0.640	1.558	9.179E-02
74	2689986	*LOC100048331*	10.9138	0.1351	10.2741	0.0511	0.640	1.558	1.552E-03
75	3123441	*OTTMUSG00000007855*	11.6389	0.2728	11.0081	0.3050	0.631	1.548	5.578E-02
76	2742557	*Bcl2l1*	8.9598	0.5473	8.3295	0.1079	0.630	1.548	1.220E-01
77	2732961	*Mff*	10.2530	0.2997	9.6288	0.1780	0.624	1.541	3.615E-02
78	1228534	*Fech*	11.4882	0.5271	10.8670	0.2157	0.621	1.538	1.319E-01
79	1245717	*Epb4.9*	9.6578	0.1658	9.0386	0.0792	0.619	1.536	4.297E-03
80	1223437	*Gnas*	9.3254	0.4820	8.7073	0.1782	0.618	1.535	1.056E-01
81	2536676	*LOC383981*	14.0264	0.4834	13.4096	0.1255	0.617	1.533	9.919E-02
82	1231718	*1810027O10Rik*	13.0472	0.1569	12.4307	0.1688	0.616	1.533	9.785E-03
83	1258682	*Map2k2*	10.7572	0.0886	10.1442	0.1188	0.613	1.529	2.010E-03
84	2719498	*2700055A20Rik*	11.2060	0.2506	10.5939	0.2178	0.612	1.528	3.313E-02
85	2684205	*4930431B09Rik*	10.6637	0.3649	10.0534	0.1256	0.610	1.527	5.195E-02
86	2550089	*2010001F03Rik*	11.4219	0.3122	10.8123	0.1595	0.610	1.526	3.947E-02

87	2597030	*Smox*	13.0159	0.2549	12.4085	0.0698	0.607	1.524	1.638E-02
88	2939503	*Ttc39a*	9.6160	0.2541	9.0100	0.0964	0.606	1.522	1.812E-02
89	2710731	*Fech*	10.4295	0.5433	9.8240	0.3346	0.606	1.522	1.756E-01
90	2477324	*Trim10*	9.1685	0.7541	8.5643	0.1257	0.604	1.520	2.429E-01
91	1250752	*Knsl5*	10.2142	0.2997	9.6140	0.0265	0.600	1.516	2.594E-02
92	1218380	*Hist2h2ac*	12.2579	0.1184	11.6603	0.2141	0.598	1.513	1.337E-02
93	2695008	*Ncoa4*	11.3031	0.7025	10.7058	0.2862	0.597	1.513	2.443E-01
94	2647885	*Bcl2l1*	8.8706	0.7009	8.2752	0.0651	0.595	1.511	2.168E-01
95	1219904	*Tspan33*	11.7388	0.1691	11.1451	0.1542	0.594	1.509	1.088E-02
96	1228630	*Psmd4*	13.4905	0.0375	12.9003	0.1433	0.590	1.505	2.312E-03
97	1225372	*Tsc1*	10.0412	0.0725	9.4560	0.0547	0.585	1.500	3.661E-04
98	2628188	*Fgfr1op2*	8.1632	0.0477	8.7493	0.1361	-0.586	-1.501	2.148E-03
99	2719202	*Hist1h2bf*	9.8002	0.7070	10.3884	0.2952	-0.588	-1.503	2.544E-01
100	1240381	*Plp1*	8.5815	0.4493	9.1699	0.1319	-0.588	-1.504	9.515E-02
101	1257575	*Cst3*	11.5501	0.1951	12.1427	0.3560	-0.593	-1.508	6.477E-02
102	2981167	*Ifit2*	8.3334	0.0461	8.9281	0.0723	-0.595	-1.510	2.759E-04
103	2780205	*Ctnna1*	9.3237	0.1664	9.9229	0.2526	-0.599	-1.515	2.651E-02
104	2431807	*2610024E20Rik*	9.0752	0.1729	9.6785	0.1085	-0.603	-1.519	6.889E-03
105	2742592	*Hist1h2be*	9.8197	0.5963	10.4280	0.3595	-0.608	-1.524	2.048E-01
106	1221564	*Ltbp1*	8.3024	0.2824	8.9129	0.2117	-0.610	-1.527	4.011E-02
107	2762944	*Ifi27*	10.2634	0.1931	10.8776	0.3627	-0.614	-1.531	6.076E-02
108	1219717	*Sort1*	8.7696	0.1896	9.3841	0.1569	-0.615	-1.531	1.240E-02
109	2637624	*1810055E12Rik*	8.2040	0.0169	8.8199	0.0940	-0.616	-1.532	3.660E-04
110	1252995	*Hist1h2be*	10.5749	0.4578	11.1910	0.3286	-0.616	-1.533	1.312E-01
111	2733179	*Aldh2*	12.4886	0.5356	13.1051	0.4290	-0.617	-1.533	1.947E-01
112	2840082	*P2ry12*	9.5380	0.6190	10.1613	0.0990	-0.623	-1.540	1.601E-01
113	1239765	*Prdx5*	10.1813	0.0840	10.8070	0.2086	-0.626	-1.543	8.529E-03
114	2546073	*Wdr68*	8.2600	0.1392	8.8857	0.0175	-0.626	-1.543	1.512E-03
115	1237208	*Lamp1*	9.4342	0.1305	10.0615	0.1946	-0.627	-1.545	9.755E-03
116	2946466	*Bin1*	10.2007	0.5967	10.8304	0.2866	-0.630	-1.547	1.748E-01
117	2731735	*Ear2*	9.2701	0.2490	9.9010	0.7466	-0.631	-1.549	2.373E-01
118	1231492	*Itga6*	8.0642	0.1105	8.6954	0.1391	-0.631	-1.549	3.531E-03
119	2732229	*Prdx3*	7.9979	0.1076	8.6296	0.0046	-0.632	-1.549	5.283E-04
120	1250195	*Ndrg1*	8.9151	0.4948	9.5479	0.2487	-0.633	-1.551	1.189E-01
121	2458275	*Ubap1*	9.4274	0.4923	10.0610	0.2137	-0.634	-1.551	1.103E-01
122	2622804	*Sh3bgrl2*	8.9118	0.4941	9.5467	0.2125	-0.635	-1.553	1.104E-01
123	2590950	*Agtrap*	8.0937	0.0717	8.7338	0.0973	-0.640	-1.558	7.844E-04
124	2687905	*Msi2*	8.3328	0.0570	8.9750	0.2477	-0.642	-1.561	1.191E-02
125	1235499	*Pros1*	8.8024	0.3963	9.4476	0.2794	-0.645	-1.564	8.251E-02
126	1230137	*Tpi1*	9.5938	0.3570	10.2407	0.2419	-0.647	-1.566	6.013E-02
127	2842137	*Ilk*	10.4440	0.4787	11.0950	0.3619	-0.651	-1.570	1.335E-01
128	2492403	*Cd151*	9.1555	0.5744	9.8094	0.2786	-0.654	-1.573	1.507E-01
129	2514292	*Zyx*	12.5377	0.4208	13.1956	0.4135	-0.658	-1.578	1.256E-01
130	2750864	*2210013O21Rik*	9.4150	0.4138	10.0732	0.2131	-0.658	-1.578	7.049E-02
131	2645208	*Arhgef3*	10.3761	0.3512	11.0344	0.1133	-0.658	-1.578	3.656E-02

132	2712986	*Chi3l3*	9.0728	0.0833	9.7314	0.2256	-0.659	-1.579	9.016E-03
133	2757966	*Cxcl4*	13.5809	0.7345	14.2412	0.2195	-0.660	-1.580	2.100E-01
134	1226017	*2610035D17Rik*	7.8684	0.1393	8.5308	0.1684	-0.662	-1.583	6.299E-03
135	2684855	*Gpx4*	11.5126	0.3916	12.1758	0.1442	-0.663	-1.584	5.124E-02
136	2652181	*Mast2*	8.9298	0.2566	9.5938	0.1379	-0.664	-1.584	1.684E-02
137	1247377	*Mpeg1*	9.8823	0.0992	10.5470	0.5911	-0.665	-1.585	1.271E-01
138	2595200	*1110012O05Rik*	10.4346	0.5198	11.1033	0.2152	-0.669	-1.590	1.086E-01
139	2950957	*Rtn4ip1*	8.4451	0.1783	9.1157	0.1650	-0.671	-1.592	8.771E-03
140	1246770	*Ybx3*	8.7271	0.0456	9.4036	0.2005	-0.676	-1.598	4.685E-03
141	2458274	*Ubap1*	9.4088	0.5923	10.0854	0.1933	-0.677	-1.598	1.331E-01
142	2710905	*S100a8*	12.9048	0.4031	13.5826	0.1711	-0.678	-1.600	5.519E-02
143	2538422	*Adap1*	8.1268	0.1656	8.8076	0.2745	-0.681	-1.603	2.124E-02
144	2747754	*Pygb*	8.4850	0.2183	9.1689	0.1684	-0.684	-1.606	1.269E-02
145	1250279	*Serpinb6a*	9.3718	0.6622	10.0563	0.3103	-0.685	-1.607	1.803E-01
146	2592823	*Cdc42ep5*	8.9789	0.5241	9.6651	0.1753	-0.686	-1.609	9.791E-02
147	2836710	*Cd151*	9.1602	0.5446	9.8464	0.3940	-0.686	-1.609	1.518E-01
148	1233187	*LOC100048299*	10.7643	0.4122	11.4549	0.1689	-0.691	-1.614	5.495E-02
149	1232762	*Atox1*	9.6402	0.4374	10.3369	0.1716	-0.697	-1.621	6.209E-02
150	2700233	*Ccng2*	8.2620	0.1820	8.9647	0.0518	-0.703	-1.628	3.003E-03
151	1234812	*Dap*	10.9603	0.7642	11.6649	0.3346	-0.705	-1.630	2.174E-01
152	1258526	*Lgals3bp*	8.8086	0.1111	9.5179	0.1312	-0.709	-1.635	2.029E-03
153	2419138	*Vcl*	9.0342	0.3557	9.7451	0.2759	-0.711	-1.637	5.218E-02
154	2640560	*Gp6*	8.6053	0.4417	9.3166	0.2025	-0.711	-1.637	6.431E-02
155	1232435	*Mmrn1*	8.3081	0.2839	9.0209	0.2437	-0.713	-1.639	2.995E-02
156	1242399	*Hist1h2bc*	12.1589	0.4955	12.8755	0.1647	-0.717	-1.643	7.624E-02
157	2952914	*Ywhaz*	11.4152	0.0953	12.1368	0.0274	-0.722	-1.649	2.284E-04
158	2646166	*Ndrl*	8.6214	0.2293	9.3451	0.0937	-0.724	-1.651	7.176E-03
159	1252157	*A330102K04Rik*	13.5734	0.9188	14.2979	0.6078	-0.724	-1.652	3.183E-01
160	2645255	*Stx7*	8.7248	0.1562	9.4516	0.1456	-0.727	-1.655	4.143E-03
161	1225422	*LOC100048807*	8.7581	0.4868	9.4896	0.1066	-0.731	-1.660	6.382E-02
162	1224768	*Ehd4*	9.8930	0.5214	10.6293	0.3095	-0.736	-1.666	1.033E-01
163	1219686	*Esd*	10.3885	0.4719	11.1303	0.0428	-0.742	-1.672	5.343E-02
164	3155180	*Itpr2*	9.6937	0.1954	10.4394	0.2164	-0.746	-1.677	1.142E-02
165	1248713	*F5*	8.6214	0.3168	9.3699	0.0739	-0.748	-1.680	1.633E-02
166	2712280	*Ormdl3*	8.9933	0.3808	9.7463	0.3691	-0.753	-1.685	6.972E-02
167	3160863	*Dusp23*	9.2086	0.6830	9.9642	0.2304	-0.756	-1.688	1.436E-01
168	2990616	*Birc2*	9.7443	0.5443	10.5005	0.1839	-0.756	-1.689	8.481E-02
169	2515363	*Tpm4*	12.0100	0.6358	12.7699	0.2713	-0.760	-1.693	1.296E-01
170	2469294	*Bicd2*	9.9262	0.3332	10.6884	0.2974	-0.762	-1.696	4.171E-02
171	2650106	*6330578E17Rik*	9.8499	0.3653	10.6176	0.2887	-0.768	-1.703	4.611E-02
172	2591082	*Nsep1*	10.8548	0.2178	11.6232	0.1511	-0.768	-1.703	7.377E-03
173	2759484	*C3*	8.7098	0.1640	9.4801	0.6437	-0.770	-1.706	1.150E-01
174	3161289	*Gnaz*	8.8505	0.4783	9.6228	0.3457	-0.772	-1.708	8.606E-02
175	2622801	*Sh3bgrl2*	9.0326	0.5866	9.8125	0.2177	-0.780	-1.717	9.699E-02
176	2663613	*Itgb5*	9.0924	0.5609	9.8731	0.2064	-0.781	-1.718	8.644E-02

177	2598374	*Ccdc92*	8.7812	0.3893	9.5627	0.3246	-0.782	-1.719	5.578E-02
178	1251233	*Gng11*	10.0192	0.6797	10.8141	0.1654	-0.795	-1.735	1.204E-01
179	3001598	*Ptpn11*	10.5486	0.4101	11.3470	0.3227	-0.798	-1.739	5.699E-02
180	3120510	*Gvin1*	10.4878	0.3719	11.2866	0.6584	-0.799	-1.740	1.413E-01
181	1241827	*Slc44a1*	8.2615	0.1153	9.0653	0.2054	-0.804	-1.746	4.101E-03
182	2751935	*Sytl4*	8.6709	0.3722	9.4819	0.3566	-0.811	-1.754	5.271E-02
183	2744603	*Gchfr*	9.6141	0.6956	10.4303	0.3433	-0.816	-1.761	1.425E-01
184	2694569	*LOC631037*	9.7117	0.2940	10.5284	0.1441	-0.817	-1.761	1.244E-02
185	3005873	*Sort1*	8.7744	0.1551	9.5921	0.1303	-0.818	-1.763	2.202E-03
186	1248811	*Ctla2b*	10.4414	0.7016	11.2602	0.1472	-0.819	-1.764	1.190E-01
187	1216746	*B2m*	14.4432	0.2181	15.2755	0.3463	-0.832	-1.781	2.439E-02
188	2753096	*Zfand3*	9.8994	0.4076	10.7369	0.2072	-0.837	-1.787	3.378E-02
189	2499264	*4933428A15Rik*	8.8469	0.3696	9.6894	0.2089	-0.842	-1.793	2.637E-02
190	2625351	*Sh3bgrl3*	11.6962	0.4012	12.5402	0.2307	-0.844	-1.795	3.424E-02
191	2688236	*Atp2a3*	9.8691	0.4051	10.7155	0.1226	-0.846	-1.798	2.573E-02
192	2616565	*Slc2a3*	10.2500	0.5174	11.1118	0.3165	-0.862	-1.817	6.961E-02
193	2693403	*Ela1*	9.2674	0.6268	10.1292	0.2428	-0.862	-1.817	9.054E-02
194	1230546	*Clic4*	9.1779	0.5320	10.0534	0.2529	-0.876	-1.835	6.168E-02
195	2718662	*Myl9*	9.3668	0.8261	10.2562	0.3640	-0.889	-1.852	1.631E-01
196	1225158	*1110003E01Rik*	11.0285	0.2075	11.9184	0.0655	-0.890	-1.853	2.095E-03
197	2801683	*Dstn*	8.9351	0.1108	9.8283	0.1998	-0.893	-1.857	2.482E-03
198	2939681	*Lyzs*	12.0444	0.1746	12.9403	0.4344	-0.896	-1.861	2.952E-02
199	2988143	*Plac8*	10.8462	0.1791	11.7442	0.5383	-0.898	-1.864	5.181E-02
200	2739999	*B2m*	14.3519	0.2851	15.2541	0.3380	-0.902	-1.869	2.415E-02
201	2806159	*Tmsb4x*	14.6266	0.6830	15.5480	0.1729	-0.921	-1.894	8.618E-02
202	2936671	*Skap2*	10.2520	0.2965	11.1744	0.2190	-0.922	-1.895	1.231E-02
203	3117381	*Fhl1*	9.2981	0.6969	10.2344	0.2760	-0.936	-1.914	9.651E-02
204	2966722	*Ywhah*	12.0501	0.8297	12.9973	0.3968	-0.947	-1.928	1.490E-01
205	2682800	*Cox5a*	10.2564	0.2319	11.2073	0.1506	-0.951	-1.933	3.987E-03
206	2687547	*Sdpr*	9.1087	0.6505	10.0639	0.3364	-0.955	-1.939	8.678E-02
207	2631093	*Cd81*	10.7176	0.3331	11.6746	0.3012	-0.957	-1.941	2.099E-02
208	1218347	*Mylk*	10.2077	0.7622	11.1696	0.3703	-0.962	-1.948	1.207E-01
209	2746283	*Mrvi1*	8.6849	0.4854	9.6577	0.3212	-0.973	-1.963	4.435E-02
210	2771380	*Cd52*	8.3865	0.4565	9.3603	0.2329	-0.974	-1.964	3.019E-02
211	1252076	*Lyz2*	10.2350	0.4266	11.2214	0.2998	-0.986	-1.981	3.059E-02
212	1245845	*A530055J02Rik*	7.7973	0.0277	8.7844	0.1266	-0.987	-1.982	1.909E-04
213	2842601	*Gp9*	10.2792	0.8559	11.2756	0.3335	-0.996	-1.995	1.335E-01
214	2725414	*Cd9*	9.7728	0.8312	10.7740	0.3086	-1.001	-2.002	1.222E-01
215	2699531	*Rgs10*	11.9318	0.7077	12.9373	0.2596	-1.005	-2.008	8.200E-02
216	2734340	*Stx11*	11.1232	0.8572	12.1387	0.3175	-1.016	-2.022	1.267E-01
217	2878071	*Lyz*	14.0839	0.0375	15.1021	0.4954	-1.018	-2.025	2.380E-02
218	2674575	*Mmd*	11.2576	0.8966	12.2936	0.2648	-1.036	-2.051	1.274E-01
219	1249498	*Plek*	10.2776	0.6284	11.3178	0.2050	-1.040	-2.057	5.267E-02
220	1236465	*F2rl2*	8.1269	0.0825	9.1746	0.2743	-1.048	-2.067	3.176E-03
221	1226555	*5430417L22Rik*	9.8597	0.6286	10.9108	0.2308	-1.051	-2.072	5.306E-02

222	2907793	*Nptn*	12.4020	0.7841	13.4558	0.2791	-1.054	-2.076	9.337E-02
223	2713285	*Fhl1*	9.3013	0.7018	10.3633	0.3706	-1.062	-2.088	8.134E-02
224	1228102	*Ppbp*	10.6549	1.0629	11.7412	0.2450	-1.086	-2.123	1.596E-01
225	2665490	*Litaf*	10.9058	0.8011	12.0022	0.2267	-1.096	-2.138	8.471E-02
226	2616309	*Pttg1ip*	11.4681	0.7800	12.5788	0.4606	-1.111	-2.160	1.009E-01
227	2855315	*Hist1h1c*	11.2302	0.5430	12.3574	0.3281	-1.127	-2.184	3.702E-02
228	1219471	*Mylk*	9.3789	0.6359	10.5163	0.3178	-1.137	-2.200	5.026E-02
229	2653205	*Gp1bb*	9.4816	0.8179	10.6210	0.3180	-1.139	-2.203	8.775E-02
230	2613908	*Alox12*	11.8218	0.9986	12.9655	0.3984	-1.144	-2.210	1.392E-01
231	2761109	*Clic4*	11.2222	0.8243	12.3756	0.4195	-1.153	-2.224	9.690E-02
232	2954868	*Oasl2*	10.2000	0.5292	11.3953	0.8012	-1.195	-2.290	9.732E-02
233	2855310	*Mpl*	9.1397	0.5518	10.3531	0.3863	-1.213	-2.319	3.552E-02
234	1246808	*Serpine2*	8.5707	0.3748	9.7998	0.3948	-1.229	-2.344	1.739E-02
235	2523169	*Treml1*	10.6343	0.8678	11.8864	0.3716	-1.252	-2.382	8.319E-02
236	3161372	*AU023871*	9.9055	0.8380	11.2534	0.3782	-1.348	-2.545	6.402E-02
237	2519673	*Vwf*	9.7469	0.7749	11.1276	0.4640	-1.381	-2.604	5.712E-02
238	2908435	*Ppbp*	12.7856	0.9772	14.1747	0.3658	-1.389	-2.619	8.241E-02
239	2730100	*Cib3*	7.7474	0.0976	9.1422	0.2396	-1.395	-2.630	7.324E-04
240	2727153	*LOC100046120*	10.5224	0.9927	11.9364	0.4158	-1.414	-2.665	8.521E-02
241	2774537	*Hist1h1c*	11.4908	0.5157	12.9316	0.3161	-1.441	-2.715	1.454E-02
242	1253819	*Prkar2b*	9.7177	0.4990	11.2201	0.1257	-1.502	-2.833	7.195E-03
243	2467007	*2810484G07Rik*	11.2090	0.9766	12.7177	0.2887	-1.509	-2.846	6.225E-02
244	1236762	*Gp5*	9.6844	0.7649	11.3673	0.3295	-1.683	-3.211	2.490E-02
245	2450735	*Rn18s*	13.6123	0.9507	15.6042	0.7135	-1.992	-3.978	4.400E-02
246	1218799	*Emb*	7.8593	0.0961	9.9252	0.1289	-2.066	-4.187	2.414E-05
247	2615468	*Fgfr1op2*	8.3577	0.0660	11.3018	0.0878	-2.944	-7.696	1.287E-06

Differentially expressed transcripts between knockout and wild type mice were identified by calculating the $\log_2$- and fold-changes (FC) of the averaged expression values. *P*-values were calculated by 1-way analysis of variance (ANOVA). Only transcripts with fold-changes of ≥1.5 and ≤-1.5 are shown.
