# Supplementary material for: Maps of Open Chromatin Guide the Functional Follow-Up of Genome-Wide Association Signals: Application to Hematological Traits
Source: PLoS Genet. 2011 Jun 30;7(6):e1002139. doi: 10.1371/journal.pgen.1002139 (PMC3128100; doi:10.1371/journal.pgen.1002139)
Supplement: Table S8 — Functional ontology classification of differentially expressed genes between Pik3cg−/− and wild-type mice. (PDF) [file pgen.1002139.s015.pdf]

Table S8. Functional ontology classification of differentially expressed genes between *Pik3cg*$^{-/-}$ and wild type mice.

#	GO term	Biological process	*P*-value	Sample frequency	Background frequency	Genes
1	0009987	Cellular process	3.86E-16	123/187 (65.8%)	11382/33954 (33.5%)	*Tsc1, Rgs10, Tmsb4x, Cst3, Ybx1, Psmd4, Ywhah, Snx15, Fech, Myo6, Map2k2, Acp1, Hist2h2ac, Smox, Atpif1, Ccng2, Gng11, Hist1h1c, Cdkn2c, Glrx5, Bicd2, Clic4, Cela1, C3, Lyz2, Cul4a, Gpx4, Rnf10, Cd81, Sh3bgrl3, Ifit2, Csda, Atp2a3, Car2, Cap1, Bcl2l1, Stx7, Fzr1, Skap2, Dstn, Sorl1, Cmas, Ctnna1, Prdx3, Chka, Acsl1, Ilk, Sytl4, Msi2, Alox12, Nptn, Arhgef3, Vwf, Atox1, Birc2, Actb, F2rl2, Serpine2, Rnf11, Dusp23, Slc44a1, Sdpr, Itpr2, Itgb5, Rffl, Ptp4a3, Pabpc1, Ywhaz, Ifi30, Zyx, Mmd, Plp1, St3gal5, Prdx5, Tpi1, Ehd4, Fancl, Lyz1, Sort1, Gnaz, Fis1, Litaf, Prkar2b, Cux1, Treml1, Lgals3bp, Gp9, Nusap1, Fhl1, F5, Pros1, Ranbp10, Emb, P2ry12, Urod, Gnas, St6galnac2, Itga6, Cdc42ep5, Cd9, Trim10, Arf5, Gp1bb, Snx3, Pygb, Mylk, Dap, Bin1, Epb4.1, Stx11, Spnb1, Ptpn11, Agtrap, Slc2a3, G3bp2, Mast2, Epb4.9, Plek, Ndrg1, Gp5, Pnpo, E2f2, Vcl*
2	0065008	Regulation of biological quality	2.64E-14	41/187 (21.9%)	1503/33954 (4.4%)	*Tsc1, Tmsb4x, Fech, Myo6, Glrx5, C3, Rnf10, Cd81, Sh3bgrl3, Car2, Dstn, Prdx3, Ilk, Sytl4, Alox12, Nptn, Gp6, Vwf, Atox1, F2rl2, Serpine2, Ywhaz, Ifi30, Plp1, Prdx5, Treml1, Gp9, F5, Pros1, P2ry12, Cdc42ep5, Cd9, Trim10, Gp1bb, Epb4.1, Spnb1, Ptpn11, Agtrap, Epb4.9, Plek, Gp5*
3	0007596	Blood coagulation	7.84E-12	13/187 (7.0%)	91/33954 (0.3%)	*C3, Gp6, Vwf, F2rl2, Serpine2, Treml1, Gp9, F5, Pros1, P2ry12, Gp1bb, Plek, Gp5*
4	0007599	Hemostasis	9.09E-12	13/187 (7.0%)	92/33954 (0.3%)	*C3, Gp6, Vwf, F2rl2, Serpine2, Treml1, Gp9, F5, Pros1, P2ry12, Gp1bb, Plek, Gp5*
5	0050817	Coagulation	1.21E-11	13/187 (7.0%)	94/33954 (0.3%)	*C3, Gp6, Vwf, F2rl2, Serpine2, Treml1, Gp9, F5, Pros1, P2ry12, Gp1bb, Plek, Gp5*

6	0065007	Biological regulation	3.71E-10	84/187 (44.9%)	7128/33954 (21.0%)	*Tsc1, Rgs10, Tmsb4x, Cst3, Ybx1, Psmd4, Ywhah, Fech, Myo6, Map2k2, Atpif1, Ccng2, Gchfr, Gng11, Cdkn2c, Glrx5, Cela1, C3, Cul4a, Gpx4, Rnf10, Cd81, Sh3bgrl3, Csda, Car2, Bcl2l1, Fzr1, Skap2, Dstn, Ctnna1, Prdx3, Ilk, Sytl4, Alox12, Nptn, Gp6, Arhgef3, Vwf, Atox1, Birc2, F2rl2, Serpine2, Sdpr, Itpr2, Ywhaz, Ifi30, Plp1, Prdx5, Ehd4, Fancl, Gnaz, Litaf, Prkar2b, Cux1, Treml1, Lgals3bp, Gp9, Nusap1, F5, Pros1, Ranbp10, P2ry12, Gnas, B2m, Itga6, Cdc42ep5, Cd9, Trim10, Arf5, Gp1bb, Ctla2b, Bin1, Epb4.1, Spnb1, Ptpn11, Agtrap, Slc2a3, G3bp2, Mast2, Epb4.9, Plek, Gp5, E2f2, Vcl*
7	0042060	Wound healing	3.06E-09	13/187 (7.0%)	143/33954 (0.4%)	*C3, Gp6, Vwf, F2rl2, Serpine2, Treml1, Gp9, F5, Pros1, P2ry12, Gp1bb, Plek, Gp5*
8	0050878	Regulation of body fluid levels	1.01E-08	13/187 (7.0%)	157/33954 (0.5%)	*C3, Gp6, Vwf, F2rl2, Serpine2, Treml1, Gp9, F5, Pros1, P2ry12, Gp1bb, Plek, Gp5*
9	0071840	Cellular component organization or biogenesis	3.22E-08	42/187 (22.5%)	2396/33954 (7.1%)	*Tsc1, Tmsb4x, Ywhah, Fech, Myo6, Hist2h2ac, Ccng2, Gchfr, Hist1h1c, C3, Lyz2, Gpx4, Cd81, Cap1, Bcl2l1, Fzr1, Dstn, Sorl1, Ctnna1, Ilk, Alox12, Nptn, Birc2, Actb, Serpine2, Pf4, Ywhaz, Ehd4, Lyz1, Sort1, Nusap1, Pros1, Ranbp10, Itga6, Cd9, Bin1, Epb4.1, Spnb1, Ptpn11, Epb4.9, Plek, Vcl*
10	0006950	Response to stress	5.03E-08	31/187 (16.6%)	1371/33954 (4.0%)	*Cst3, Psmd4, Chi3l3, Fech, Cela1, C3, Lyz2, Cul4a, Gpx4, Fzr1, Prdx3, Gp6, Vwf, Atox1, Birc2, F2rl2, Serpine2, Ywhaz, Fancl, Lyz1, Treml1, Gp9, F5, Pros1, P2ry12, B2m, Cdc42ep5, Gp1bb, Ptpn11, Plek, Gp5*
11	0050896	Response to stimulus	5.20E-08	45/187 (24.1%)	2752/33954 (8.1%)	*Ifi27l2a, Cst3, Ybx1, Psmd4, Chi3l3, Fech, Myo6, S100a8, Cela1, C3, Lyz2, Cul4a, Gpx4, Ifit2, Bcl2l1, Fzr1, Prdx3, Acsl1, Alox12, Gp6, Vwf, Atox1, Birc2, F2rl2, Serpine2, Pf4, Ywhaz, Oasl2, Fancl, Lyz1, Sort1, Prkar2b, Treml1, Gp9, F5, Pros1, P2ry12, Gnas, B2m, Itga6, Cdc42ep5, Gp1bb, Ptpn11, Plek, Gp5*
