# Supplementary material for: Maps of Open Chromatin Guide the Functional Follow-Up of Genome-Wide Association Signals: Application to Hematological Traits
Source: PLoS Genet. 2011 Jun 30;7(6):e1002139. doi: 10.1371/journal.pgen.1002139 (PMC3128100; doi:10.1371/journal.pgen.1002139)
Supplement: Table S9 — Genetic loci selected for the high-density DNA tiling array. (PDF) [file pgen.1002139.s016.pdf]

Table S9. Genetic loci selected for the high-density DNA tiling array.

**(A) Association loci**									
**#**	**Gene(s)**	**GWA index SNP(s)**		**Trait(s)**	**Reference(s)**	**Genomic position**			**Interval (kb)**
		**ID**	**Position**			**Chromosome**	**Start**	**End**	
1	*PCSK9*	rs11206510	55,268,627	Early-onset MI	[1]	1p32.3	55,240,000	55,300,000	60.00
2	*VAV3*[b]	rs17229705	107,940,484	PLS	[2]	1p13.3	107,890,000	108,055,000	165.00
3	*CELSR2, PSRC1, SORT1*	rs599839	109,623,689	CAD	[3,4]	1p13.3	109,489,481	109,739,481	250.00
		rs646776	109,620,053	Early-onset MI	[1]				
4	*PEAR1*[b]	rs3737224	155,146,204	PLS	[2]	1q23.1	155,100,000	155,250,000	150.00
5	*FCER1G*[a,b]	rs3557	159,455,517	PLS	[2]	1q23.3	159,441,711	159,465,662	23.95
6	*DNM3*	rs10914144	170,216,373	MPV	[5]	1q24.3	170,130,000	170,390,000	260.00
7	*TMCC2*	rs1668873	203,502,613	MPV	[5]	1q32.1	203,445,000	203,540,000	95.00
8	*MIA3*	rs17465637	220,890,152	CAD / early-onset MI	[1,3]	1q41	220,678,228	221,078,228	400.00
		rs3008621	220,870,669	CAD	[4]				
9	*EHD3*	rs647316	31,318,333	MPV	[5]	2p23.1	31,305,000	31,346,000	41.00
10	*STK39*[b]	rs6749447	168,749,632	SBP / DBP	[6]	2q24.3	168,695,000	168,800,000	105.00
		rs3754777	168,724,160						
11	*WDR12*[a]	rs6725887	203,454,130	Early-onset MI	[1]	2q33.1	203,443,575	203,494,639	51.06
12	*gene desert*	rs2943634	226,776,324	CAD	[3,4]	2q36.3	226,532,739	226,982,739	450.00
13	*ITPR1*[b]	rs17786144	4,804,575	PLS	[2]	3p26.2	4,760,000	4,860,000	100.00
14	*RAF1*[a,b]	rs3729931	12,601,516	PLS	[2]	3p25.1	12,590,108	12,690,678	100.57
15	*ULK4*	rs9815354	41,887,655	DBP	[7,8]	3p22.1	41,710,000	41,970,000	260.00
16	*ARHGEF3*	rs12485738	56,840,816	MPV	[5,9]	3p14.3	56,750,000	56,880,000	130.00
17	*MRAS*	rs9818870	139,604,812	CAD / MI	[10]	3q22.3	139,550,000	139,613,000	63.00
18	*P2RY12*[b]	rs1472122	152,517,292	PLS	[2]	3q25.1	152,410,000	152,580,000	170.00
19	*ITGA2*[b]	rs41305896	52,311,475	PLS	[2]	5q11.2	52,267,000	52,436,366	169.37
20	*PHACTR1*	rs12526453	13,035,530	Early-onset MI	[1]	6p24.1	12,985,000	13,060,000	75.00

21	*HFE*	rs1800562	26,201,120	MCV	[5]	6p22.1	26,190,000	26,260,000	70.00
22	*BAK1*	rs210135	33,648,670	PLT	[5]	6p21.31	33,644,000	33,665,000	21.00
23	*MAPK14*[b]	rs851007	36,172,014	PLS	[2]	6p21.31	36,050,000	36,210,000	160.00
24	*BSYL, CCND3*	rs11970772	42,033,268	MCV	[5]	6p21.1	41,985,000	42,105,000	120.00
25	*HBS1L, MYB*	rs9402686	135,469,510	MCV	[5]	6q23.3	135,400,000	135,582,003	182.00
26	*MTHFD1L*	rs6922269	151,294,678	CAD	[3,4,11]	6q25.1	151,199,579	151,399,579	200.00
27	*SLC22A3, LPAL2, LPA*	rs2048327–rs3127599–rs7767084–rs10755578 haplotype		CAD	[12]	6q25.3–q26	160,780,000	161,055,000	275.00
28	*CD36*[b]	rs1049654	80,113,391	PLS	[2]	7q21.11	80,045,000	80,137,000	92.00
29	*TFR2*	rs7385804	100,073,906	RBC	[5]	7q22.1	100,050,000	100,100,000	50.00
30	*FLJ36031, PIK3CG*	rs342293	106,159,455	MPV	[5,13]	7q22.3	106,085,000	106,190,000	105.00
31	*AK3, RCL1, JAK2* [(b, for PLS)]	rs385893	4,753,176	PLT	[5]	9p24.1	4,730,000	5,050,000	320.00
		rs10429491	5,040,706	PLS	[2]				
32	*CDKN2A, CDKN2B*	rs1333049	22,115,503	CAD / MI	[1,3,4,11,14]	9p21.3	21,900,000	22,200,000	300.00
		rs4977574	22,088,574	Early-onset MI	[1]				
33	*CACNB2*	rs11014166	18,748,804	DBP	[7,8]	10p12.33	18,570,000	18,840,000	270.00
34	*CXCL12/SDF1*	rs501120	44,073,873	CAD	[3,4]	10q11.21	43,950,000	44,200,000	250.00
		rs1746048	44,095,830	Early-onset MI	[1]				
35	*JMJD1C*[a]	rs2393967	64,803,162	MPV	[5]	10q21.2–q21.3	64,586,991	64,905,728	318.74
36	*CYP17A1, C10orf32*	rs1004467	104,584,497	SBP	[7,8]	10q24.32	104,520,000	104,690,000	170.00
37	*BET1L, SIRT3, PSMD13*	rs11602954	192,856	MPV	[5]	11p15.5	180,000	240,000	60.00
38	*PLEKHA7*	rs381815	16,858,844	SBP	[7,8]	11p15.1	16,795,000	16,960,000	165.00
39	*ATP2B1*	rs2681492	88,537,220	SBP	[7,8]	12q21.33	88,460,000	88,650,000	190.00
		rs2681472	88,533,090	HYP / DBP	[7,8]				
40	*SH2B3/LNK, ATXN2*	rs3184504	110,368,991	MI; SBP / DBP	[7,8,15]	12q24.12	110,310,000	110,570,000	260.00
		rs11065987	110,556,807	PLT; CAD / MI	[5]				
41	*PTPN11*[a]	rs11066301	111,355,755	PLT; CAD / MI	[5]	12q24.13	111,330,919	111,442,100	111.18

42	*TBX3, TBX5*	rs2384550	113,837,114	DBP	[7,8]	12q24.21	113,810,000	113,930,000	120.00
43	*HNF1A/TCF1, C12orf43*	rs2259816	119,919,970	CAD / MI	[10]	12q24.31	119,850,000	119,980,000	130.00
44	*WDR66*	rs7961894	120,849,966	MPV	[5,9]	12q24.31	120,760,000	120,970,000	210.00
45	*TPM1*	rs11071720	61,129,049	MPV	[5]	15q22.2	61,080,000	61,160,000	80.00
46	*SMAD3*[b]	rs17228212	65,245,693	CAD	[3]	15q22.33–q23	65,150,000	65,350,000	200.00
47	*CSK, ULK3*	rs6495122	72,912,698	DBP	[7,8]	15q24.1	72,810,000	73,030,000	220.00
48	*CDH13*	rs11646213	81,200,152	HYP / SBP / DBP	[16]	16q23.3	81,190,000	81,240,000	50.00
49	*MAP2K4*[a,b]	rs41307923	11,968,409	PLS	[2]	17p12	11,854,860	11,997,776	142.92
50	*TAOK1*	rs2138852	24,727,475	MPV	[5,9]	17q11.2	24,680,000	24,930,000	250.00
51	*GSDMA, ORMDL3*	rs17609240	35,364,215	WBC	[5]	17q12	35,314,374	35,387,545	73.17
52	*CD226*	rs893001	65,667,825	MPV	[5]	18q22.2	65,620,000	65,730,000	110.00
53	*MAP2K2*[b]	rs350916	4,045,775	PLS	[2]	19p13.3	4,025,000	4,095,000	70.00
54	*LDLR*	rs1122608	11,024,601	Early-onset MI	[1]	19p13.2	11,010,000	11,120,000	110.00
55	*AKT2*[b]	rs41275750	45,429,935	PLS	[2]	19q13.2	45,380,000	45,520,000	140.00
56	*APOC1, APOC4, APOE*[b]	rs4420638	50,114,786	CAD	[11]	19q13.32	50,000,000	50,230,000	230.00
57	*GP6*[a,b]	rs1613662	60,228,407	PLS	[2]	19q13.42	60,206,885	60,251,444	44.56
58	*SIRPA*	rs6136489	1,871,734	MPV	[5]	20p13	1,820,000	1,950,000	130.00
59	*SLC5A3, MRPS6, KCNE2*	rs9982601	34,520,998	Early-onset MI	[1]	21q22.11	34,340,000	34,530,000	190.00
60	*GNAZ*[b]	rs3788337	21,742,017	PLS	[2]	22q11.22	21,720,000	21,840,000	120.00
61	*FBXO7*	rs9609565	31,197,528	MCV	[5]	22q12.3	31,186,000	31,250,000	64.00
62	*TMPRSS6*	rs5756506	35,797,338	MCH	[5]	22q12.3	35,730,000	35,830,000	100.00

(B) Lineage-specific reference genes					
**Lineage**	**Gene**	**Genomic position**			**Interval (kb)**
		**Chromosome**	**Start**	**End**	
**monocytic**	*ASGR2*	17p13.1	6,943,365	6,960,852	17.49
	CD163	12p13.31	7,512,676	7,549,681	37.01
	FER1L3	10q23.33	95,054,176	95,234,029	179.85
	KLF4	9q31.2	109,284,956	109,293,576	8.62
	PID1	2q36.3	229,594,933	229,846,301	251.37
	RIN2	20p11.23	19,816,210	19,933,100	116.89
	SLC46A2	9q32	114,679,021	114,694,866	15.85
	TMEM176A	7q36.1	150,126,787	150,135,141	8.35
**erythroblastoid**	*CA1*	8q21.2	86,425,709	86,479,594	53.89
	CALB2	16q22.3	69,948,127	69,983,842	35.72
	EPB42	15q15.2	41,274,720	41,302,773	28.05
	ERAF	16p11.2	31,444,704	31,449,625	4.92
	FAM83A	8q24.13	124,261,933	124,293,499	31.57
	GDF15	19p13.11	18,355,968	18,362,986	7.02
	HBZ	16p13.3	140,854	146,504	5.65
	LOC51252	2q11.2	96,903,349	96,929,558	26.21
**megakaryocytic**	*ADCY6*	12q13.12	47,444,248	47,471,087	26.84
	CMTM5	14q11.2	22,913,857	22,920,821	6.96
	DDEF2	2p25.1	9,262,345	9,465,257	202.91
	LY6G6D	6p21.33	31,789,112	31,795,560	6.45
	MEIS1	2p14	66,514,036	66,655,395	141.36
	MYLK	3q21.1	124,811,833	125,087,839	276.01
	NFIB	9p23–p22.3	14,069,847	14,305,945	236.10
	SELP	1q24.2	167,822,711	167,868,001	45.29

Genomic coordinates are based on the human reference genome, build hg18 (NCBI build 36). Abbreviations: CAD: coronary artery disease; MI: myocardial infarction; MPV: mean platelet volume; PLT: platelet counts; PLS: platelet signaling; WBC: white blood cell counts; RBC: red blood cell counts; MCV: mean corpuscular volume of erythrocytes; MCH: mean corpuscular hemoglobin; SBP: systolic blood pressure; DBP: diastolic blood pressure; HYP: hypertension. Key: [a]target gene ± 10 kb; [b]biological evidence for association.

References:

1. Myocardial Infarction Genetics Consortium (2009) Genome-wide association of early-onset myocardial infarction with single nucleotide polymorphisms and copy number variants. Nat Genet 41: 334-340.

2. Jones CI, Bray S, Garner SF, Stephens J, de Bono B, et al. (2009) A functional genomics approach reveals novel quantitative trait loci associated with platelet signalling pathways. Blood 114: 1405-1416.
3. Samani NJ, Erdmann J, Hall AS, Hengstenberg C, Mangino M, et al. (2007) Genome-wide association analysis of coronary artery disease. New Engl J Med 357: 443-453.
4. Coronary Artery Disease Consortium (2009) Large scale association analysis of novel genetic loci for coronary artery disease. Arterioscl Throm Vas Biol 29: 774-780.
5. Soranzo N, Spector TD, Mangino M, Kühnel B, Rendon A, et al. (2009) A genome-wide meta-analysis identifies 22 loci associated with eight hematological parameters in the HaemGen consortium. Nat Genet 41: 1182-1190.
6. Wang Y, O'Connell JR, McArdle PF, Wade JB, Dorff SE, et al. (2009) Whole-genome association study identifies STK39 as a hypertension susceptibility gene. Proc Natl Acad Sci USA 106: 226-231.
7. Newton-Cheh C, Johnson T, Gateva V, Tobin MD, Bochud M, et al. (2009) Genome-wide association study identifies eight loci associated with blood pressure. Nat Genet 41: 666-676.
8. Levy D, Ehret GB, Rice K, Verwoert GC, Launer LJ, et al. (2009) Genome-wide association study of blood pressure and hypertension. Nat Genet 41: 677-687.
9. Meisinger C, Prokisch H, Gieger C, Soranzo N, Mehta D, et al. (2009) A genome-wide association study identifies three loci associated with mean platelet volume. Am J Hum Genet 84: 66-71.
10. Erdmann J, Grohennig A, Braund PS, König IR, Hengstenberg C, et al. (2009) New susceptibility locus for coronary artery disease on chromosome 3q22.3. Nat Genet 41: 280-282.
11. The Wellcome Trust Case Control Consortium (2007) Genome-wide association study of 14,000 cases of seven common diseases and 3,000 shared controls. Nature 447: 661-678.
12. Trégouët D-A, König IR, Erdmann J, Munteanu A, Braund PS, et al. (2009) Genome-wide haplotype association study identifies the SLC22A3-LPAL2-LPA gene cluster as a risk locus for coronary artery disease. Nat Genet 41: 283-285.
13. Soranzo N, Rendon A, Gieger C, Jones CI, Watkins NA, et al. (2009) A novel variant on chromosome 7q22.3 associated with mean platelet volume, counts and function. Blood 113: 3831-3837.
14. Schunkert H, Götz A, Braund P, McGinnis R, Trégouët D-A, et al. (2008) Repeated replication and a prospective meta-analysis of the association between chromosome 9p21.3 and coronary artery disease. Circulation 117: 1675-1684.
15. Gudbjartsson DF, Bjornsdottir US, Halapi E, Helgadottir A, Sulem P, et al. (2009) Sequence variants affecting eosinophil numbers associate with asthma and myocardial infarction. Nat Genet 41: 342-347.
16. Org E, Eyheramendy S, Juhanson P, Gieger C, Lichtner P, et al. (2009) Genome-wide scan identifies CDH13 as a novel susceptibility locus contributing to blood pressure determination in two European populations. Hum Mol Genet 18: 2288-2296.
